# North American avian species that migrate in flocks show greater long-term non-breeding range shift rates

**DOI:** 10.1186/s40462-024-00527-0

**Published:** 2025-01-13

**Authors:** Stephen H. Vickers, Timothy D. Meehan, Nicole L. Michel, Aldina M. A. Franco, James J. Gilroy

**Affiliations:** 1 School of Environmental Sciences, University of East Anglia, Norwich, NR4 7TJ UK; 2 National Audubon Society, 225 Varick Street, New York, NY 10014 USA

**Keywords:** Migration, Distributions, Range dynamics, Navigation, Social behaviour, Cultural inheritance

## Abstract

**Background:**

Many species are exhibiting range shifts associated with anthropogenic change. For migratory species, colonisation of new areas can require novel migratory programmes that facilitate navigation between independently-shifting seasonal ranges. Therefore, in some cases range-shifts may be limited by the capacity for novel migratory programmes to be transferred between generations, which can be genetically and socially mediated.

**Methods:**

Here we used 50 years of North American Breeding Bird Survey and Audubon Christmas Bird Count data to test the prediction that breeding and/or non-breeding range-shifts are more prevalent among flocking migrants, which possess a capacity for rapid social transmission of novel migration routes.

**Results:**

Across 122 North American bird species, social migration was a significant positive predictor for the magnitude of non-breeding centre of abundance (COA) shift within our study region (conterminous United States and Southern Canada). Across a subset of 81 species where age-structured flocking was determined, migrating in mixed-age flocks produced the greatest shifts and solo migrants the lowest. Flocking was not a significant predictor of breeding COA shifts, which were better explained by absolute population trends and migration distance.

**Conclusions:**

Our results suggest that social grouping may play an important role in facilitating non-breeding distributional responses to climate change in migratory species. We highlight the need to gain a better understanding of migratory programme inheritance, and how this influences spatiotemporal population dynamics under environmental change.

**Supplementary Information:**

The online version contains supplementary material available at 10.1186/s40462-024-00527-0.

## Background

Species persistence in changing environments can be limited by the speed at which populations spread to new areas [23, 44, 84]. Migratory organisms face a unique challenge in this context: colonisation of new breeding or non-breeding sites can require not only successful dispersal, but also changes in the distance and/or direction of migratory movements that link newly-colonised areas with other seasonally-occupied ranges [27, 61]. Thus, whilst individuals may reach suitable new migratory destinations as a result of both intrinsic and extrinsic factors, population-scale establishment of novel routes can only occur if subsequent generations are able to repeat the successful journey taken by those pioneer individuals [82]. The mechanisms by which migration programs are inherited are thus integral to migratory range dynamics [82], but these remain poorly understood [16, 38, 74, 77].

Migratory navigation has a strong genetic basis in many species [12, 19, 50, 57, 79]. Directional migration requires at least a capacity to orientate (i.e., a compass) and evaluate distance travelled [46], many migrants are thought to achieve this through genetically-inherited programmes, utilizing a range of visual, magnetic, and olfactory cues [33, 57]. Species that migrate in groups, by contrast, also have the potential for social route learning from experienced conspecifics or even heterospecifics [21, 31, 45, 54, 55, 76]. Social grouping is widespread across migratory taxa [1, 10], and there is evidence that both movement timing and direction are strongly influenced by observational and collective social interactions in many species [51, 60].

**Fig. 1:**
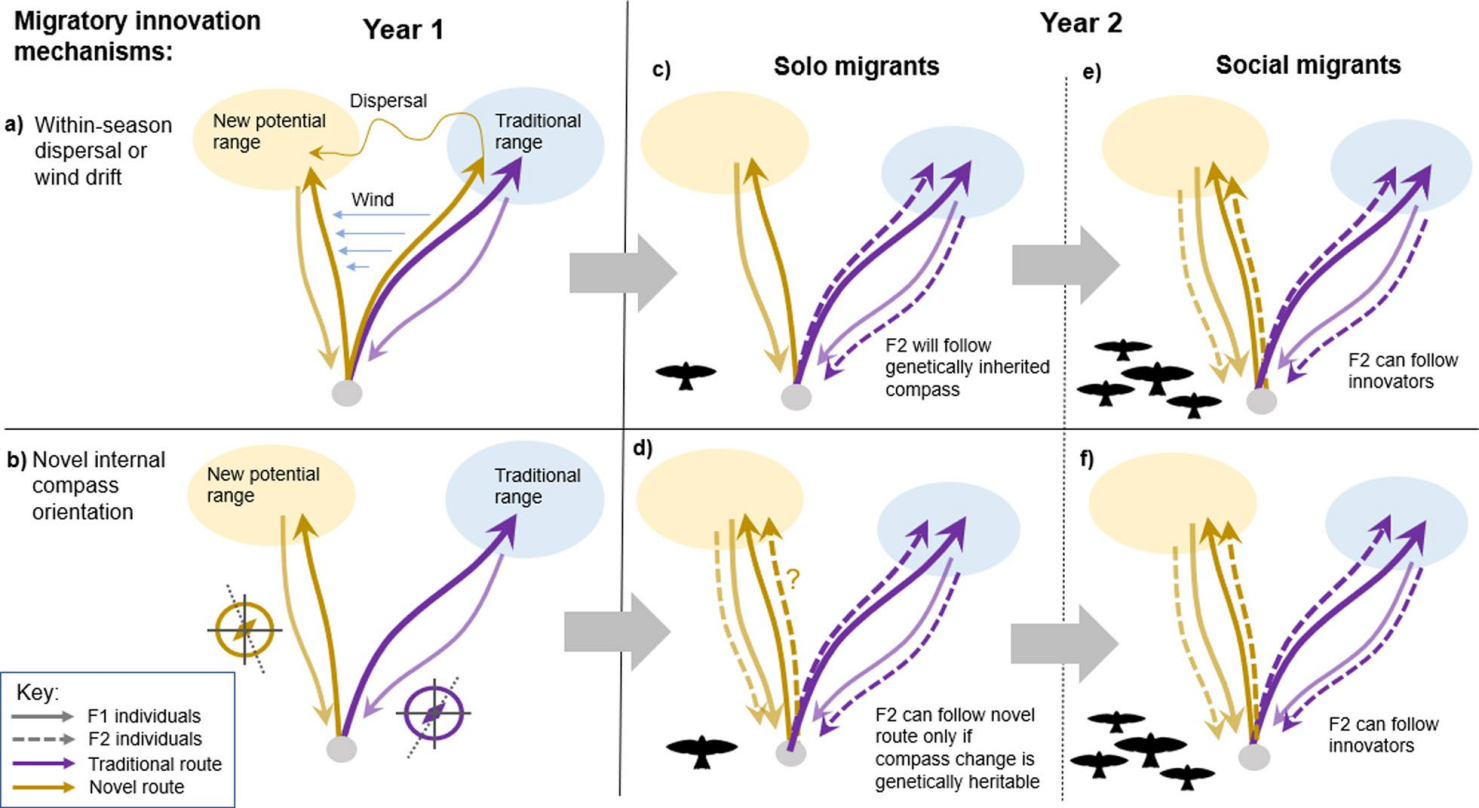
Pathways of seasonal range colonisation in migratory species. Migratory innovation can occur when individuals or groups make novel movements (F1), which can arise from (**a**) exogenous mechanisms (e.g., drift by winds or geomagnetic anomalies), exploratory dispersal within a seasonal stage, or abmigration (following other species), as well as (**b**) through the emergence of novel endogenous navigation programmes. Successful colonisation depends on their offspring (F2) or other conspecifics repeating the novel migration route in subsequent years. In solitary migrants (**c**, **d**), innovations are unlikely to be transferred if they originate from stochastic drift or dispersal, as F2 individuals will continue to follow inherited programmes to the original range (**c**). Novel endogenous navigation programmes can similarly only be transferred to F2 in solitary migrants if they are genetically heritable (**d**). In social migrants (**e**, **f**), by contrast, route innovations can be transferred between generations if F2 individuals follow returning F1 innovators on return migrations, regardless of whether the initial innovation mechanism was stochastic **e** or endogenous **f**

Novel migration route establishment has been documented both through heritable changes in endogenous migration programmes (e.g., [11, 28]), and also through socially-mediated changes in behaviour (e.g., [55, 76]). The relative prevalence of these mechanisms in driving migratory change remains unclear, though the pace of socially-transmitted behavioural change is known to outstrip that of genetic adaptation in some circumstances [3]. Here, we hypothesise that species exhibiting social migration will show greater propensities for novel route establishment than solitary migrants thus facilitating faster range-shift and/or colonisation rates, as social migration offers more pathways for migratory innovations to be transferred from one generation to the next (Fig. 1). Whilst non-social (solitary) migrants might be more prone to individual vagrancy events (i.e., occurrence outside the normal geographic range or migration route) than social migrants, for example due to a higher likelihood of navigation errors or susceptibility to drift [10, 49], we predict that successful colonisation will be less frequent in these species, as this is only likely to occur when there is strong genetic heritability of the new migratory programme (Fig. 1c and d).

While we hypothesise social migration will allow for greater range shift rates, we also expect this effect to be somewhat seasonally dependent. This is due to a reduced reliance on heritable changes in migratory programmes for breeding range shifts compared to non-breeding range shifts as shown in Vickers et al. [82]. This is because novel breeding sites (arrived at by within-season movements or changes in migratory movements) can plausibly be successfully colonized in the absence of heritable changes in migratory programmes, provided that future offspring exhibit natal philopatry (as is typical in migratory species [24]) and their existing migratory programmes still carry them to viable non-breeding areas. In effect, offspring need only return to their previously experienced natal location (e.g., via ‘magnetic maps’ [53]), whereas reaching a novel non-breeding location can require mechanisms of information transfer between generations (i.e., heritable changes in genetic programme or social learning). However, it is notable that Madsen et al. [55] recently documented social information being an important key driver of rapid colonisation of new breeding ranges in an Arctic-breeding goose, suggesting that flocking can help facilitate breeding range-shifts in some cases.

To test these predictions, we characterised migratory flocking for 122 North American avian species and examined how this explains variation in rates of distributional change. Migratory species display huge variation in the magnitude of recent range-shifts, and the fundamental drivers of this variation have remained elusive [5, 27, 47, 65]. Flocking behaviour can take many forms, and the potential for social route innovation could vary with characteristics such as age-structured phenology, flock size (as larger flocks may have greater capacity for information transfer [4], and greater navigation accuracy [8, 9, 68]), and nocturnal versus diurnal migration [1]. We test whether these flocking characteristics correlate with rates of shift in breeding and/or non-breeding centres of abundance within our study region (conterminous United States and southern Canada) as a proxy of range shifts, whilst controlling for other species characteristics which may influence their capacity to colonise new areas. For example, partial (only a portion of individuals migrate) vs. complete (all individuals migrate) migratory species, as partial migrants may be able to shift their non-breeding range more rapidly through spatially biased shifts in the proportion of migrants in a population. We also control for effects of phylogeny, population trend, specialism, generation length, and range size.

## Methods

### Determining migratory flocking strategies

We classified migratory flocking strategies of North American migratory bird species using data from a range of sources including Beauchamp [8], Birds of the World [15], and Evans and O’Brien [30]. Beauchamp [8] previously collated data on migratory flock size for 180 of the 410 migratory species that breed in Canada and the conterminous USA using 420 primary literature sources. We used the maximum flock size while flying during migration values to initially classify migratory flocking as a binary variable, assigning any species with a reported maximum travelling flock size greater than 2 to be a flocking species. The presence or absence of heterospecifics within these counts was not provided, but it is reported that this distinction rarely applied to group size while flying. We augmented these data through a systematic appraisal of species accounts from Birds of the World [15], which provided binary classification of migratory flocking for 321 species. These accounts aggregate scholarly information on avian life histories using primary literature which include descriptions of migratory behaviour. 30 species were only categorised by Beauchamp [8], 171 only by Birds of the World [15], and 150 by both. Where disagreements were found between sources, Birds of the World [15] was used to classify flocking, this being the most recent literary source aggregating current literature under expert review. We undertook sensitivity testing of this migratory flocking metric, whereby we assessed the sensitivity of Beauchamp [8] and Birds of the World [15] flocking data to how flocking was defined (see Supplementary Material). For Beauchamp [8] where flocking was categorised based upon maximum recorded flock sizes whilst on migration, we assessed the effect of increasing the threshold of maximum travelling flock size to 5 and 10 from the original value of 2. For Birds of the World [15] classifications which relied upon written descriptions rather than specific flock sizes, we assessed differences between solo, small (typically < 10 individuals), and large flocks (typically > 10 individuals).

**Fig. 2:**
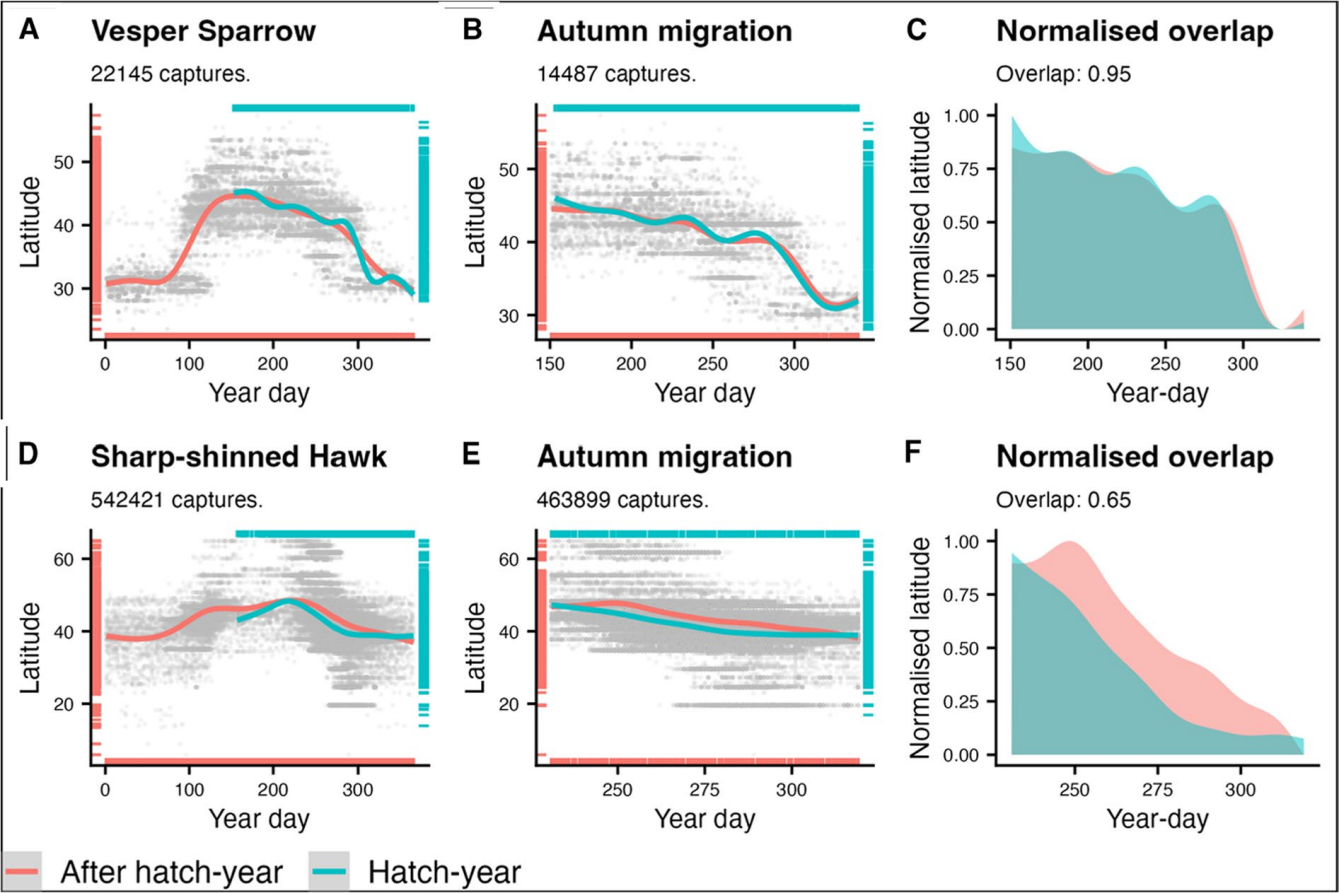
Examples of the methodology for assessing overlap in timing of migration between age cohorts using banding data, showing examples of a species with high cohort overlap and thus concurrent migration timing of age classes (Vesper Sparrow *Pooecetes gramineus*, **A**–**C**) and a species with relatively low overlap and thus non-concurrent migratory timing (Sharp-shinned Hawk Accipiter striatus, **D**–**F**). After initially fitting GAMs to latitudes of banding events across the year for hatch-year and after hatch-year age cohorts of a given species across all USGS banding events 1960–2019 (**A** and **D**), we restricted the dataset to the autumn migration period, assessed as the temporal region where latitude shows a clear negative trend and refit the GAM models (southward migration; **B** and **E**). We then normalised the GAM-predicted mean latitudes for each age class to a 0–1 scale and calculated the overlap in area under the curve as an index of cohort temporal overlap during migration (**C** and **F**)

To further classify whether species typically migrate in mixed-age or age-separated flocks, we used a combination of Birds of the World [15] and USGS banding data to evaluate the extent of age-structured migration phenology across all the flocking species. For 155 of the 321 migratory species of which binary classification of migratory flocking was available, information on migratory cohort timing was reported in Birds of the World [15] species accounts. We expanded this information to a wider species pool through analysis of USGS banding data 1960–2019 [25] for species that had at least 1000 banding records during this period. Banding data allowed quantification of cohort timing for 145 species, providing an additional 34 classifications within the final pool of analysed species. To analyse cohort timing using banding data, we assessed change in the latitude of banding events through the migratory period. As a species undertakes migration, we see a latitudinal shift in their distribution (unless the migration is entirely longitudinal, which is rare), and for species that migrate in an age- or sex-structured manner this shift can be separated temporally. To assess this, we fitted generalised additive models (GAM,package ‘mgcv’, [88]) for each species whereby the latitude of banding events was explained by a thin-plate regression spline term of Julian day and its interaction with age class (hatch-year and after hatch-year), in effect fitting a separate smoothed line of best fit for each age-class. In each model the default basis dimension (k) value of − 1 was used to give 10 basis functions for spline fitting, and Generalised Cross-Validation was used to estimate smoothness parameters. An initial model was fit to the entire dataset for each species to visually assess appropriate start and end dates of autumn migration in each species (i.e., a clear trend of changing latitude, Fig. 2A and D). We then fitted a second model with identical structure to this limited time period (Fig. 2 B and E), and categorised cohort timing by assessing similarity between the age-specific smoothed lines. To do this we first normalised the fitted smoothed lines for each cohort (using min–max normalisation) and calculated the proportional overlap in area under the curve between the two normalised fitted curves (Fig. 2C and F). We used a threshold of overlap greater than 0.85 to classify species as having mixed-age migration timing, as this value maximised agreement between model-derived classifications of age-structured timing and those from accounts of migration behaviour in Birds of the World [15] (Figure SM1).

We subsequently refined the resulting information on flocking and cohort timing to a single categorical variable, where ‘mixed-age flocking’ are species that typically migrate in conspecific flocks containing adults and juveniles (i.e. species with and without prior migratory experience), ‘age-separated flocking’ are those that migrate in conspecific flocks but adults and juveniles are typically separated temporally and thus do not migrate together, and finally ‘solo’ migrants are those that migrate predominantly alone and seldom or never join flocks.

### Other species traits

Our analyses accounted for a range of other traits that may influence COA shift rates (Table 1). We extracted information on migration type (complete or partial) and whether species predominantly migrate nocturnally or diurnally from Beauchamp [8], supplemented by text summaries from Birds of the World [15]. For species where no information on nocturnal/diurnal migration could be found (*n* = 7), their closest congener was used, as evolutionary transitions in migration timing of North American migrants are relatively uncommon within clades [8, 9]. To control for the potential influence of changes in overall population size on COA shifts, we took the loglinear breeding population size trend estimate (1966–2019) for each species from publicly-available USGS breeding bird survey results (see [67]). We converted this to absolute values to measure population change rather than directional trends, as both positive and negative trends may contribute to the observed magnitude of shifts in centre of abundance (COA). Whereby a growing or shrinking population is more likely to have directional range expansion or contraction, respectively. Generation length (years) was included to control for the influence of life history characteristics associated with pace of life and body size, taking values from Bird et al. [13] calculations incorporating model-estimated age of first reproduction, maximum longevity, and annual adult survival. To control for biases related to niche breadth [69], we include the species’ total range size for the relevant season on the assumption that species with broader niche breadth have a greater propensity for range shifts as previously shown across several taxa [36]. To calculate seasonal range sizes, we use eBird seasonally modelled ranges from the ebirdst R package [73], which utilise bird sightings submitted to the eBird citizen-science database alongside environmental covariates to predict occurrence probability within an ensemble modelling strategy based on the Adaptive Spatio-Temporal Exploratory Model (AdaSTEM, [32]). Where these have not been modelled for the species (breeding *n* = 4; non-breeding *n* = 4; both *n* = 3), we instead use the coarser BirdLife/Natureserv seasonal range maps which are expert-drawn rather than empirically modelled (BirdLife 2019). For many species only part of the respective range was covered by our study region, and we therefore calculated the sampled range size as the total area of each seasonal range covered by the analysis strata (Figure SM2), including this as a predictor in all analyses to account for potential biases arising from incomplete geographical sampling of species ranges. We also include migratory distance (km) as a potential predictor of COA shifts [65], which we derived from great circle centroid distances between the eBird/Birdlife/Natureserv breeding and non-breeding ranges.

**Table 1:** Migratory traits and how the data was collated

Trait	Description	Source
Migration type	Is the species a partial or complete migrant? (Yes/No)	[15]
Migration distance	Great circle distance (km) between the breeding range centroid and non-breeding centroid	Derived from [73] and [14]
Cohort timing	Do juveniles migrate before, concurrently, or after adults?	[25] (see SM2), [15]
Flocking	Does the species flock on migration? (Yes/no)	[8, 9, 15]
Flocking behaviour	Species flocking behaviour (mixed-age flocks, age-separated flocks, or solo)	Combination of cohort timing and flocking variables
Absolute population trend	Survey-wide loglinear population trend based upon Breeding Bird Survey data (%/yr 1966–2019 for the Core Survey Area). Absolute value taken	[67]
Generation length	Modelled generation length based upon age of first reproduction, maximum longevity, and annual adult survival	[13]
Migratory timing	At what time does the specie primarily migrate? (day or night)	[8, 9, 15]
Habitat specialism score	How specialised is the species to a certain habitat (0–100)	Derived from Wilman et al. [86] as maximum score across habitat classes
Diet specialism score	How specialised is the species to a dietary niche (0–100)	Derived from Wilman et al. [86] as maximum score across diet classes
Total breeding range size	Total size of the breeding range (km^2^) restricted to the Americas	Derived from [73] and [14]
Sampled breeding range size	Size of the breeding range (km^2^) covered by the analysis strata (Figure SM2)	Derived from [73] and [14]
Total non-breeding range size	Total size of the non-breeding range (km^2^) restricted to the Americas	Derived from [73] and [14]
Sampled non-breeding range size	Size of the non-breeding range (km^2^) covered by the analysis strata (Figure SM2)	Derived from [73] and [14]

We were able to derive complete trait data for 81 species, and partial data (lacking age-structured migratory flocking information) for 122 species. Where taxonomic nomenclature has been revised over time, manual revisions of taxonomic names were required to combine data from separate sources ensuring nomenclature remained consistent across datasets.

### Calculating annual centres of abundance

We estimated annual COAs for breeding and non-breeding periods for each species using Breeding Bird Survey (BBS) and Christmas Bird Count (CBC) data respectively. BBS surveys comprised > 4000 39.3 km linear routes throughout the United States and Canada (see [22] for detail), while CBC surveys comprised of > 2400 count circles across North America where all birds detected within a 24.1 km diameter circle, on a single day (between 14 December and 5 January) are counted (see [72] for detail).

Raw count metrics from these surveys may be liable to spatial sampling biases, particularly over time. We therefore use model-derived metrics at the stratum level, where strata are defined by all intersections of Bird Conservation Regions (BCRs) and political borders (i.e. state, provincial, and territorial borders) across North America following the USGS methodology [66]. Stratum-based metrics help to alleviate issues of imbalanced spatiotemporal sampling that are present in BBS and CBC surveys [42]. To further limit spatial bias, we limited analysis to strata across well-sampled BCRs 5 and 9–39 (Figure SM2). For consistency between seasonal analyses, assessment of COA shift rates was limited to species where at least part of both their breeding and non-breeding range was covered by this region. For some species, COA shifts therefore represent a proxy for partial range-shifts through expanding or contracting range limits within our study region. Such shifts are still hypothesised to be potentially limited by novel migration route establishment, however, magnitude of shifts may be biased by the extent of coverage and the spatial structure of the surveyed range. For stratum-level BBS annual indices, we used posterior median and 95th percentile values from Bayesian hierarchical GAMs (GAMYE model) provided by Smith & Edwards [70]. For stratum-level CBC annual indices, a Bayesian loglinear hierarchical model was used following the method described in Soykan et al. [72], with posterior median and 95th percentile values drawn from Meehan et al. [56].

To calculate the location of an annual centre of abundance (longitude and latitude, separately) for a species, the yearly mean of strata centroid locations (longitude and latitude) were weighted by the Bayesian posterior strata abundance indices (Eq. 1).

1 $$\begin{array}{*{20}c} {COA_{{Latitude}} = \frac{{\sum {Population\;\;Index_{{Stratum}} } \;\;*\;\;Latitude_{{Stratum}} }}{{\sum {Population\;Index_{{Stratum}} } }}} \\ {COA_{{Longitude}} = \frac{{\sum {Population\;\;Index_{{Stratum}} } \;\;*\;\;Longitude_{{Stratum}} }}{{\sum {Population\;\;Index_{{Stratum}} } }}} \\ \end{array}$$

We calculated COAs across the period 1970–2019 as this avoided early periods of both surveys where spatial sampling coverage was sparse. As Bayesian posteriors can be assumed to follow an approximately normal distribution around the median for sufficiently large samples [34], posterior standard deviation (SD) can be approximated using the 95th percentiles (Eq. 2).

2 $$SD = \frac{{Upper\;\;95th\;\;Percentile - Lower\;\;95th\;\;Percentile}}{{3.92}}$$

We used classical error propagation methods to incorporate uncertainty into COA calculations. Error around each posterior stratum abundance estimate was propagated using the first-order Taylor series method from package ‘methods’ [81].

#### Centre of abundance trends

We calculated linear trend coefficients separately for both latitudinal and longitudinal components of the annual COA metrics for each species (Eq. 3), representing estimates of annual latitudinal and longitudinal displacement in the COA, again propagating errors.

3 $$Linear\;trend\;coefficient = \frac{{\sum (Year_{n} - mean(Year)) \times (COA_{n} - mean\left( {COA} \right))}}{{\sum (Year_{n} - mean(Year))^{2} }}$$

A single vector of annual COA displacement was then calculated using Pythagorean theorem (Eq. 4), with propagated errors.

4 $$COA\;displacement = \sqrt {Latitudinal\;displacement^{2} + Longitudinal\;displacement^{2} }$$

### Trait analysis

We built phylogenetic generalised least squares (PGLS) models to test for the effects of species traits on annual centre of abundance shift rates for each season, controlling for potential phylogenetic collinearity [75]. Explanatory variables of migration type (complete or partial), migration distance (numeric), habitat specialism score (numeric), diet specialism score (numeric), absolute population trend (numeric), total seasonal range size (numeric), sampled seasonal range size (numeric), migratory timing (night or day), and generation length (numeric) were used within our global models. We weighted the response variable (species COA shift) by its reciprocal standard error (i.e. smaller standard errors and thus higher confidence in estimates produce higher weighting) to account for varying levels of uncertainty in COA shift estimates [63]. The first global model for each season included migratory flocking behaviour as a binary variable (flocking or solo) for a species pool of 122 (Model A). We then fitted a second global model for each season using a refined migratory flocking behaviour variable (mixed-age flocks, age-separated flocks, or solo) for the reduced species pool of 81 for which this trait information was available (Model B). Numeric variables were first scaled to Z-scores to ensure comparable effect coefficients.

We used the pgls.SEy function from package phytools [63] to fit the four global models (Models A and B for the breeding and non-breeding periods), incorporating uncertainty within our dependent variable using the propagated errors around each shift rate estimate. Global models were checked for collinearity between predictor variables using variance inflation factor scores, with a cutoff threshold of 5. Within non-breeding model B, with the refined migratory flocking behaviour explanatory variable, absolute population trend and total non-breeding range size had VIF values greater than 5 and were therefore removed from the model in a stepwise fashion until all variables had VIF scores less than 5. Model averaging was then conducting for each of the four global models where we considered all potential nested models within the global model. Averaging was conducted across candidate models within 2 AICc units of the candidate model with the lowest AICc value using the package MuMIn [7]. In order to limit overfitting, candidate models with fewer than 10 samples per variable were excluded from the model averaging process. Variables were considered significant when the 95th percentile confidence interval did not cross zero.

For all models, we used a consensus phylogenetic tree derived from the posterior distribution of complete trees produced by Jetz et al. [43] available at http://www.birdtree.org and provided by Holtmann et al. [40]. The tree was prepared on the basis of a Hackett backbone (Hackett tree [35],). Trees were trimmed based upon species names within our dataset for each model.

#### Sensitivity of migratory flocking variables

We tested for sensitivity within our migratory flocking behaviour variables by running further models of the same structure as those described above. To test for sensitivity within our binary flocking vs solo migrant variable we re-categorised flocking based upon maximum flock size while travelling from Beauchamp [8]. Increased thresholds of 5 and 10 to denote a flocking species were tested. Numeric values of flock size during migration were not routinely reported in Birds of the World [15] and as such this sensitivity testing was run on a subset of 73 species where this information was available from Beauchamp [8].

We also tested for an effect of flock size by categorising flocking species (with a flocking threshold of > 2 individuals) as those species that flock in small flocks (typically < 10 individuals) and those that migrate in large flocks (typically > 10 individuals). This categorisation was possible for a subset of 105 species, based on a combination of maximum flock size whilst travelling data from Beauchamp [8] and Birds of the World [15] species accounts.

All statistical analyses were performed with R 4.3.0 [62].

## Results

### Effects of flocking

From an initial pool of 410 migratory species that breed in Canada and the conterminous USA, we were able to characterise the extent of migratory flocking behaviour for 122 species (14 orders). Most species in our sample (103; 84%) predominantly migrate in flocks, though solitary migration was present across the avian phylogeny. We found large between-species variation in seasonal COA shift rates within our study region, particularly for non-breeding ranges, spanning species that remained highly static (e.g., Hermit Warbler *Setophaga occidentalis*, non-breeding COA shift 0.4 ± 3.6 km per annum) and others that showed relatively rapid COA shifts (e.g., Scissor-tailed Flycatcher *Tyrannus forficatus*, non-breeding COA shift 29.6 ± 6.2 km per annum). Both example species represent species present within our study region only at the extreme edge of their total non-breeding distributions, with the former showing little change in their extant range, and the latter displaying expansion of the extant range westward. However, observed COA shifts in species such as the Scissor-tailed Flycatcher may be somewhat biased by the spatial structure of their range. This can occur where the range is non-continuous and different population growth and/or colonisation rates are observed in each range patch.

**Fig. 3:**
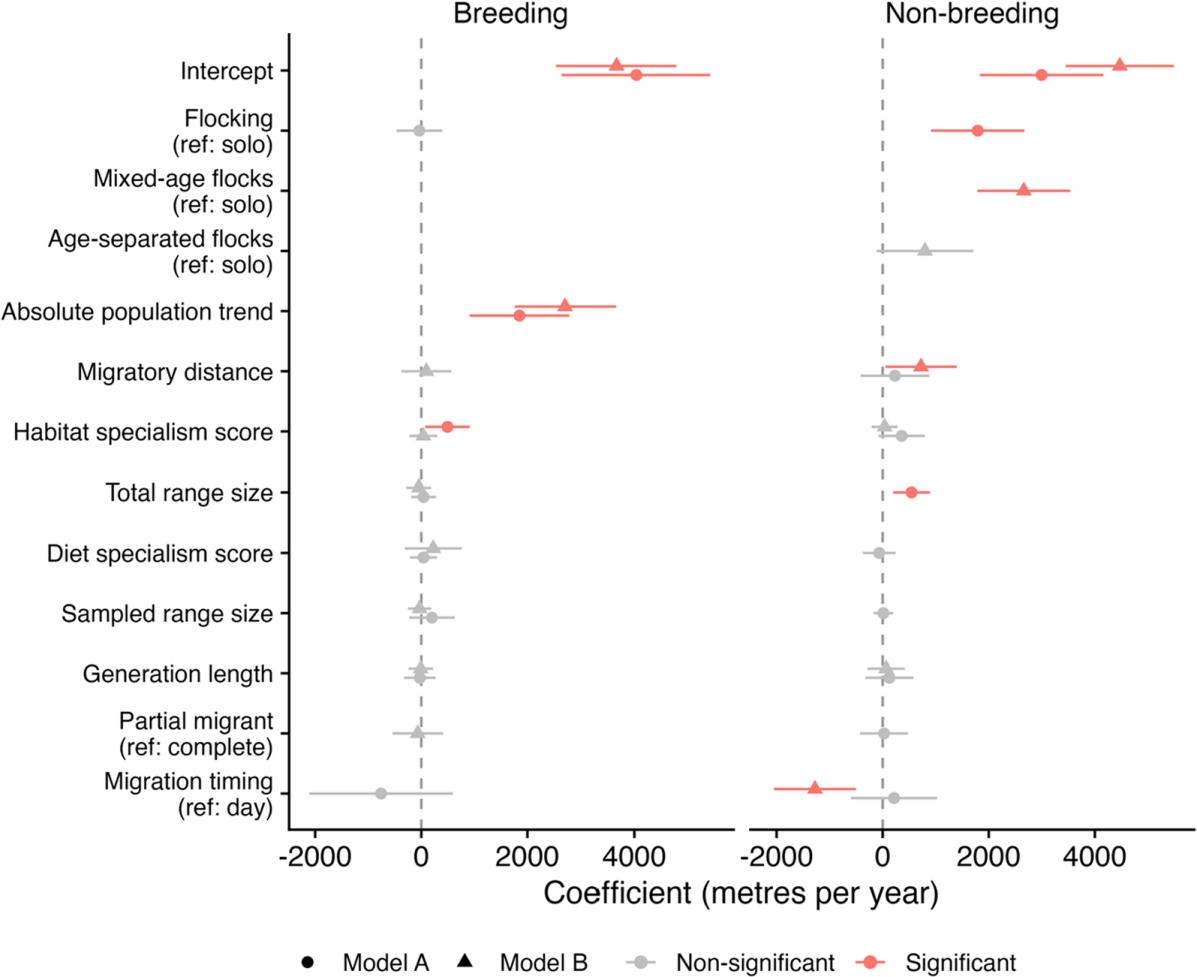
Phylogenetic Generalised Least Squares (PGLS) model coefficients for predictors of annual shift rates (metres per year) of Breeding Bird Survey (left) and Christmas Bird Count (right) centres of abundance between 1970 and 2019. Model **A** assessed migratory flocking as a binary flocking vs. solo migrants for 122 species. Model **B** refined the assessment of migratory flocking differentiating between species that flock in age-separated and mixed-age flocks, for a reduced pool of 81 species. Red points indicate significant results inferred from credible 95th percentiles (error bars) that exclude zero

Across the suite of demographic and biological traits tested across our pool of 122 species, migratory flocking had the strongest effect on COA shift rates within our study region for the non-breeding range (Fig. 3, Model A), with annual COA shift being approximately 1738 m per year higher in flocking species than those that predominantly migrate alone (Coefficient = 1738.4 ± 537.7SE, *p* = 0.001, Table SM6, Fig. 3). Species with larger total range sizes also showed significantly larger COA shifts in this model (Coefficient = 549.28 ± 177.86SE, *p* = 0.002, Table SM6, Fig. 3), but no other traits showed significant association with non-breeding COA shifts.

COA shift rates for breeding ranges within our study region, by contrast, were best predicted by absolute population trends over time, as well as habitat specialism, but were not significantly related to flocking behaviour (Fig. 3). Breeding range COA shifts were significantly greater among species that exhibited higher absolute population trends (Coefficient = 1849.15 ± 474.05SE, *p* < 0.001, Table SM2, Fig. 3), and were significantly higher among species with greater habitat specialism scores (Coefficient = 492.9 ± 212.1SE, *p* = 0.02, Table SM2, Fig. 3).

### Effects of mixed-age flocking

Within a reduced subset of 81 species (11 orders) where age-cohort migratory timing could be established, 39 species were classified as migrating in mixed-age flocks, 23 migrate within age-separated flocks, and 19 migrate solo.

**Fig. 4:**
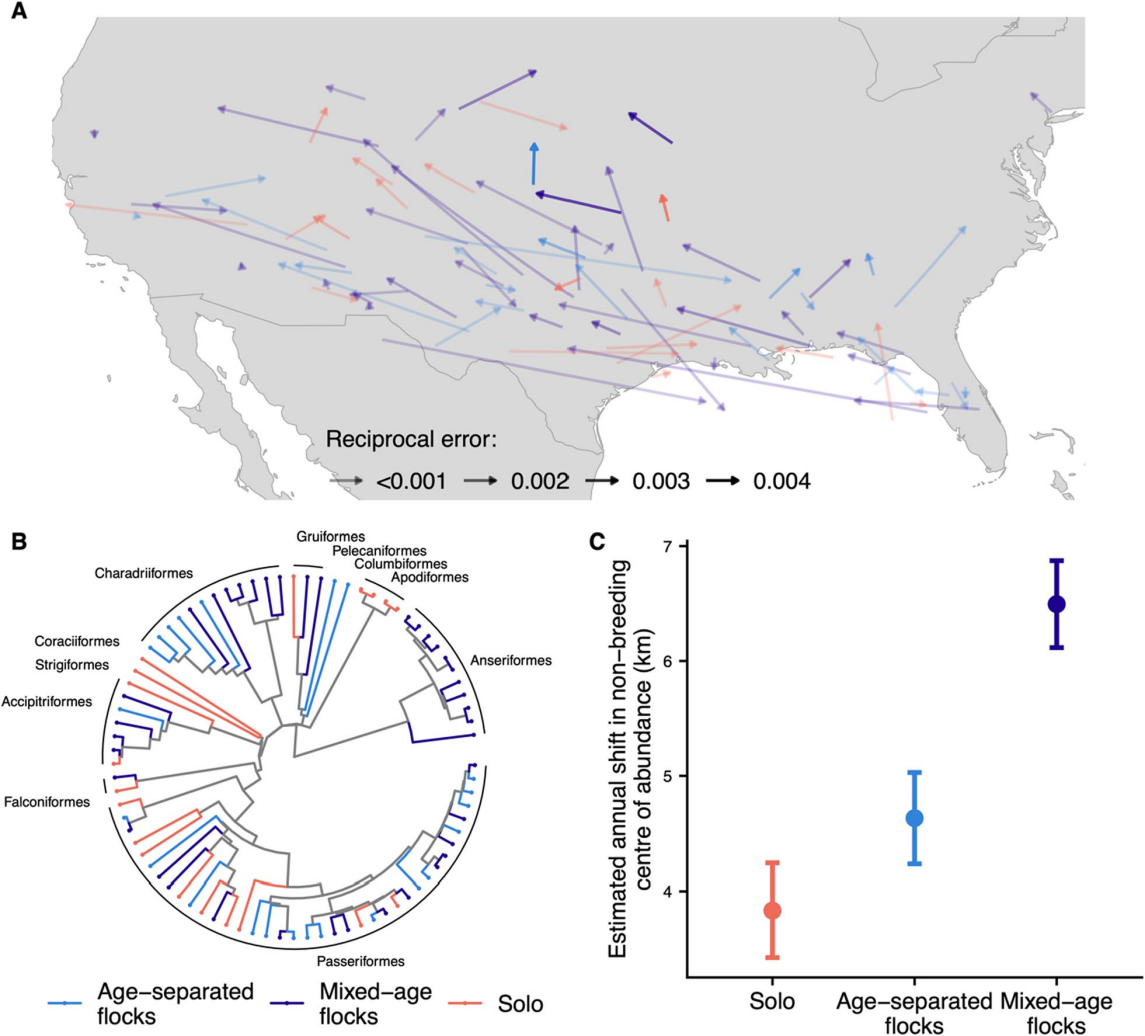
**A** Estimated shifts in non-breeding centres of abundance 1970–2019 for 81 analysed species in Model B. Arrows are coloured by migratory flocking behaviour – age-separated flocks (light-blue), mixed-age flocks (dark-blue) and solo (red) and transparency is set according to reciprocal error in estimated shift, such that more transparent arrows indicate lower confidence in estimates. **B** Phylogenetic tree of the 81 species analysed for shifts in Christmas Bird Count annual centre of abundance and incorporated into the Phylogenetic Generalised Least Squares (PGLS) model. Nodes are coloured by migratory flocking behaviour – age-separated flocks (light-blue), mixed-age flocks (dark-blue) and solo (red). **C** Estimated marginal mean annual shifts in Christmas Bird Count centre of abundance 1970–2019 for migratory flocking behaviour based upon 81 species of North American migratory birds in a Phylogenetic Generalised Least Squares (PGLS) model incorporating uncertainty in shift rates and controlling for biological and demographic traits. Error bars indicate standard error

Within this species pool, rates of non-breeding COA shift within our study region were highest in mixed-age migrating species, with annual COA shifts approximately 2660 m per annum greater than solo migrants (Coefficient = 2660.87 ± 439.18SE, *p* < 0.001, Table SM8, Figs. 3 and 4), suggestive of greater potential for naïve juveniles to learn novel migratory routes from experienced individuals in these species. For age-separated flocks, estimated COA shift rates were approximately 798 m per annum greater than solo migrants, however this difference was non-significant (Coefficient = 797.50 ± 458.94SE, *p* = 0.09, Table SM8, Figs. 3 and 4). As was the case across the full species pool, lowest estimated COA shift rates were found in species that migrate solo. These results support the prediction that within flocking species, age-cohort timing will also play an important role in range-shifting capacity. Whereby, range shifting may be more limited where the potential for social transmission of novel routes is limited due to juveniles rarely interacting with experienced individuals on migration.

We also found support for a significant effect of migratory timing on non-breeding COA shift rates, with COA shifts being approximately 1276 m per annum lower in species that primarily migrate at night (Coefficient =  − 1276.23 ± 388.50SE, *p* = 0.001, Table SM8, Fig. 3). Longer-distance migrants on the other hand showed significantly higher non-breeding shift rates in this model (Coefficient = 723.43 ± 338.13SE, *p* = 0.04, Table SM8, Fig. 3), but unlike the full species pool, we found no effect of range size. These between-sample inconsistencies suggest that these effects may be sensitive to the species pool analysed.

Breeding COA shifts within our study region in this 81 species subset were again significantly associated with absolute rates of population change (Coefficient = 2708.72 ± 475.87SE, *p* < 0.001, Table SM4, Fig. 3), but we found no significant effect of habitat specialism scores (Coefficient = 39.15 ± 133.08SE, *p* = 0.77, Table SM4, Fig. 3), indicating that this result was also sensitive to the species pool analysed. Flocking behaviour was again not significantly related to breeding COA shift rates in this sample.

### Limited effects of flock size

Across a subset of 105 species for which we could characterise typical flock size during migration, we found that species typically migrating in small flocks (typically < 10 individuals) showed significantly larger non-breeding COA shift rates than solo migrants, shifting approximately 1513 m per annum faster (Coefficient = 1513.31 ± 491.53SE, *p* = 0.002, Table SM16, Figure SM4). Species migrating in large-flocks (typically > 10 individuals) shifted approximately 2721 m per annum faster than solo migrants; however, greater uncertainty in model estimates within this group meant this difference fell short of our significance threshold (coefficient = 2721.56 ± 1506.08SE, *p* = 0.07, Table SM16, Figure SM4).

Our results were insensitive to the choice of thresholds used to define our binary flocking vs. non-flocking species (> 5 or > 10 individuals defining a flock), with effects of migratory flocking remaining broadly consistent as when flocking species were defined as those migrating in groups greater than 2 individuals (Figure SM4).

## Discussion

Across 50 years of Christmas Bird Count data, migratory flocking behaviour was a significant predictor of non-breeding centre of abundance shift rates within our study region. This pattern was evident across a broad species pool and remained after controlling for phylogenetic correlation and other morpho-ecological traits. We found no relationship between flocking and rates of breeding COA shift within our study region, however, supporting the expectation that the behavioural traits influencing shift rates may differ between the breeding and non-breeding seasons [74, 82]. Our results support the hypothesis that social behaviour during migration can play an important role in facilitating inter-generational transfer of novel migratory programmes, which may in turn influence potential rates of population-scale change in non-breeding distributions over time [55, 60, 82].

Dispersal ability is inevitably a key limiting factor in the colonisation of new ranges [20, 85], but previous work has largely overlooked the distinctions between processes operating in breeding and non-breeding ranges (i.e. reproductive versus non-reproductive dispersal, sensu [82]. The mechanisms determining non-reproductive dispersal are poorly understood, but may include itinerant sampling movements, misorientation and weather-drift during migration, and over-shooting or short-stopping along the historical migration axis (Fig. 1, [29, 74, 82]). A link between range-shift rates and migratory flocking does not necessarily suggest a greater propensity for non-reproductive dispersal behaviour in these species per se, but a greater capacity for the outcomes of these events to be transferred between generations.

In non-social migrants (i.e. ‘solo’ migrants within our analyses), the transfer of novel migratory programmes between generations is likely to have greater dependence upon heritable changes in migratory gene complexes [82], unless environmental cue-response mechanisms allow for changes within existing migratory routes such as short-stopping [87]. Our finding that solo migrants are experiencing the slowest COA shift rates suggests that these genetically-heritable migratory innovations may arise infrequently relative to socially-mediated changes, despite cases of novel migratory route emergence through genetic mutations being well documented [11, 78]. Recent evidence suggests that even simple migratory programmes have complex polygenetic underpinnings [28, 71], and thus multiple adaptive mutations may be necessary to precipitate significant population-scale changes in migratory patterns. Thus, whilst solitary migrants may often stray from traditional migration routes due to external factors such as wind drift and navigation errors, the frequency with which novel movements are transferred to subsequent generations may be limited to rarer cases where their underpinnings are genetically heritable.

The role of social learning in facilitating migration innovation, by contrast, is increasingly recognised (see e.g., [45, 58, 76]). Our results suggest that this may extend to spatial range dynamics, strengthening the evidence that social inheritance pathways are important for adaptation in migratory species [21, 55]. While our analyses are limited to birds, socially-mediated navigation has been documented in many taxa including cetaceans [18], ungulates [45], fish [54] and invertebrates [26]. The role of social interactions in aiding successful navigation is well documented [1], but the capacity for social grouping to enable plasticity in migratory patterns merits further study across these wider taxa.

Whilst our findings suggest that any form of flocking may confer increased potential for migratory range change, the nature and composition of flocking may also influence this potential. The importance of intermixing between adults and juveniles is perhaps unsurprising, as this is essential for between-generation transfer of novel innovations (Fig. 1). We also found some evidence for an effect of flock size, but high uncertainty in model estimated COA shift rates for species migrating in large flocks, alongside difficulty in accurately measuring typical migrating flock size, mean this result remains inconclusive. It may be that the more fundamental difference between species exhibiting any degree of flocking (meaning some information transfer is possible) and those that remain solitary is the most vital distinction. Social migrants also vary in the extent of group cohesion which may be more important than group size, with some species maintaining stable social or even family groups during migration, whilst others frequently change group membership [1]. In the absence of data to characterise this trait across our species pool, we were unable to consider group cohesion in our analyses. Group cohesion is often associated with strong migratory connectivity [1, 37], suggesting that this trait may reduce the frequency of itinerant movements occurring, though it may also promote successful colonisation following any such rare events.

Despite the strong effect of migratory flocking behaviour on non-breeding COA shift rates within our study region, we found no such relationship for breeding ranges. Instead, we found that changes in overall population size had the largest effect size for influencing breeding COA shift rates, which was expected given that spatially-structured abundance changes can influence COA metrics even in the absence of range expansion or contraction [52]. The lack of a significant effect of absolute population trend in non-breeding COA shifts, by contrast, suggests that non-breeding COA shifts are more indicative of colonisation-extinction dynamics, which have previously been shown to be more prevalent in non-breeding than breeding distributions [27].

For most species within our study, the seasonal range covered by our study area only represents part of the seasonal range and COA shifts here therefore represent a proxy for partial range-shifts within the study area rather than total range shift. Such partial range shifts would however still be expected to be limited by a species’ capacity to colonise new areas and therefore heritable migratory innovation (Fig. 1). For some species the sampled range was small and may only represent the extreme range edge of an otherwise large spatial range. We attempted to control for potential biases that such cases may introduce, however the size of sampled range showed no significant effect on range shift rates in either season and it is therefore unlikely that such cases had overt directional bias on our results.

Despite significant advances in species tracking [80] and technologies such as radar [59], we still lack a robust understanding of the extent and nature of migratory flocking behaviour in many species. Quantification of colonisation rates and/or range shifts can also be difficult to achieve in practice across broad species pools, particularly for species with stochastic (e.g., nomadic species) or patchy distributions (e.g., alpine specialists) [52]. Such species may also experience more species-specific barriers to range-shift not addressed here. Using COA metrics as a proxy for range shifts alleviates some of the quantification issues by focussing on the overall mass of a population,however, COA shifts remain an imperfect measure for range shift and/or colonisation rates as they can arise from multiple mechanisms, including regional changes in population abundance within a static range, rather than shifts arising from colonisation-extinction dynamics [41]. This is particularly the case for species with non-contiguous ranges within our study region, where large gaps between range patches alongside variation in patch-specific population growth rates may lead to elevated COA shift rates that do not accurately reflect colonisation rates. Furthermore, apparent low rates of range-shift do not necessarily indicate a limited capacity to colonise new areas, as suitable new areas may not be available for colonisation [17, 39] or shifts may not be required.

## Conclusions

Our results add to the growing evidence for a potential role for social learning in enabling migratory programme adaptation [21, 55, 60], which may be crucial for species exposed to seasonally-independent shifts in zones of suitable bioclimate [82]. Gaps remain in our understanding of migratory programme evolution, but significant advances are likely to come from improving our understanding of how genetic mechanisms of change interact with social mechanisms. Understanding the determinants of migratory change will have important consequences for conservation planning [2, 48], particularly in enabling more realistic forecasts of future range-shift capacity, accounting for limitations imposed by the adaptive capacity of some migratory life histories. Given that many migratory species are declining [6, 64, 83], our findings also suggest an urgent need to explore the potential for Allee effects in flock-migrating species, given the potential importance of social interactions in both maintaining migratory ontogeny and facilitating adaptation.

## Supplementary Information

Additional file 1.

## Data Availability

Datasets and code used for analysis here are available on GitHub: https://github.com/SHVickers94/Migratory_flocking_MS.
