# Supplementary material for: North American avian species that migrate in flocks show greater long-term non-breeding range shift rates
Source: Mov Ecol. 2025 Jan 13;13:3. doi: 10.1186/s40462-024-00527-0 (PMC11730467; doi:10.1186/s40462-024-00527-0)
Supplement: Supplementary file 1 — Additional file 1. [file 40462_2024_527_MOESM1_ESM.pdf]

# Supplementary material

## Species that migrate in flocks show greater long-term range shift rates

Stephen H. Vickers[1*], Timothy D. Meehan[2], Nicole L. Michel[2], Aldina M.A. Franco[1] and James J. Gilroy[1]

[1] School of Environmental Sciences, University of East Anglia, NR4 7TJ Norwich, UK.

[2] National Audubon Society, 225 Varick Street, New York, NY, 10014 USA.

[*] Corresponding author: svickers@rvc.ac.uk/sjedwards94@hotmail.co.uk

*SM1. Migratory timing banding analysis*

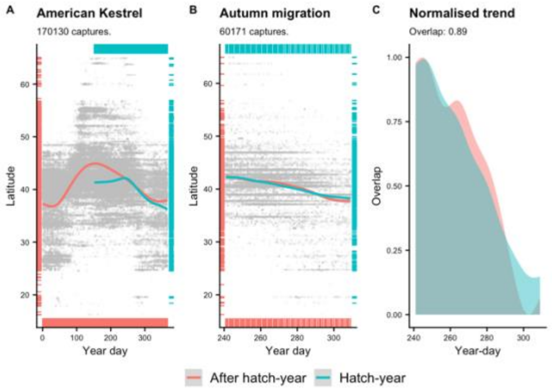


BOTW classification: Concurrent

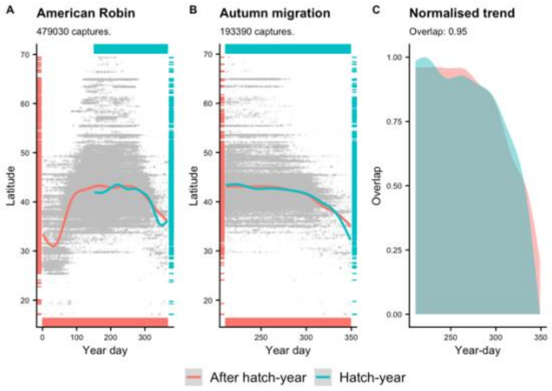


BOTW classification: Not classified

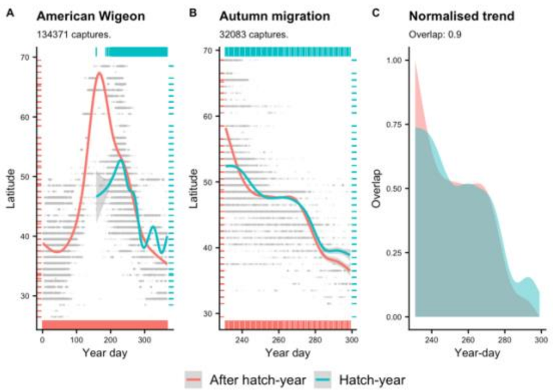


BOTW classification: Concurrent

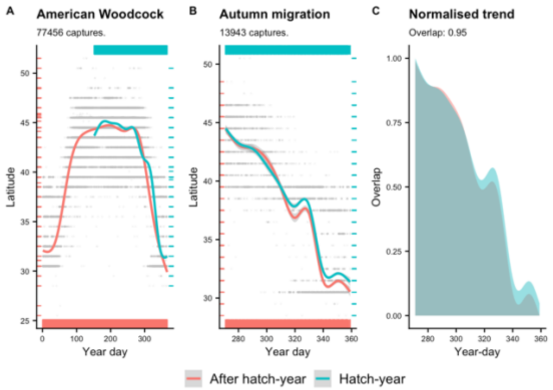


BOTW classification: Not classified

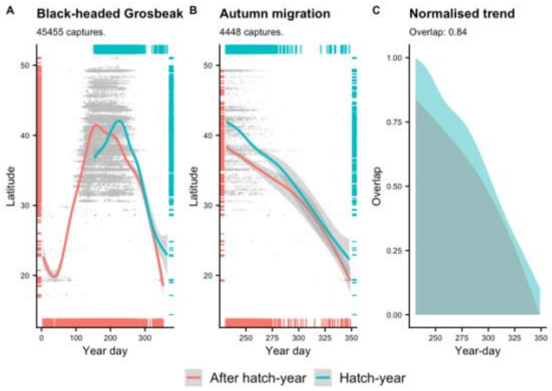


BOTW classification: Not classified

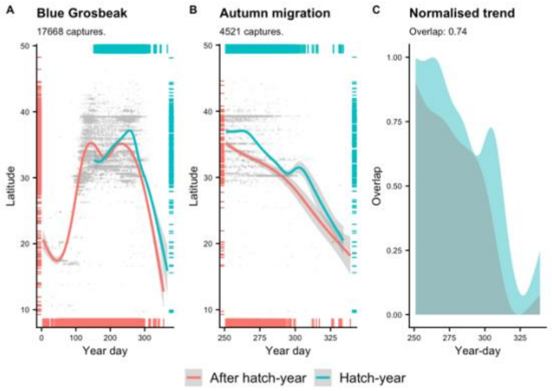


BOTW classification: Not classified

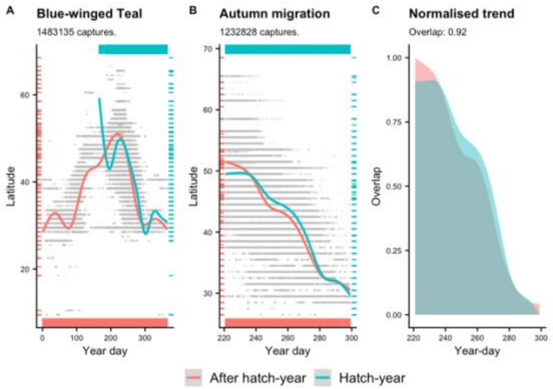


BOTW classification: Concurrent

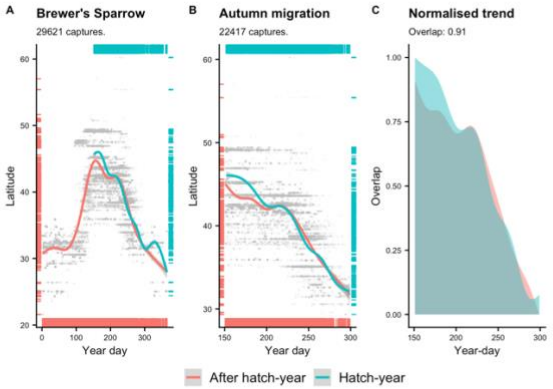


BOTW classification: Not classified

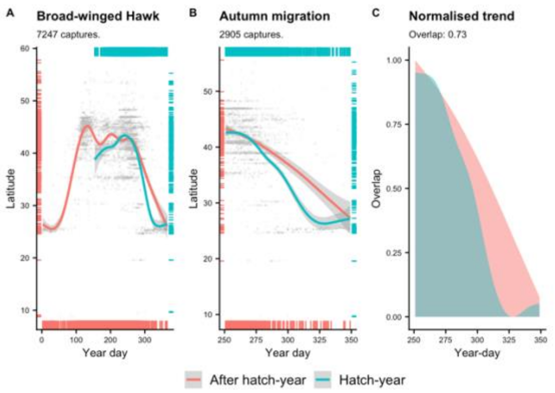


BOTW classification: Concurrent

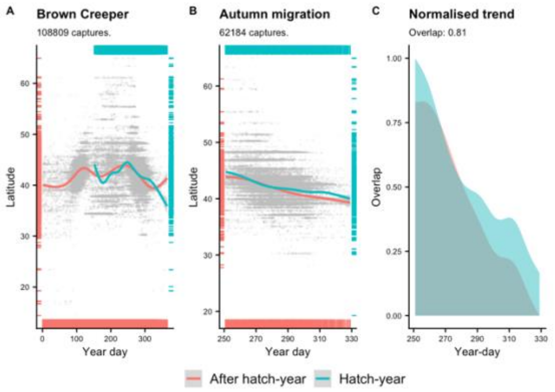


BOTW classification: Not classified

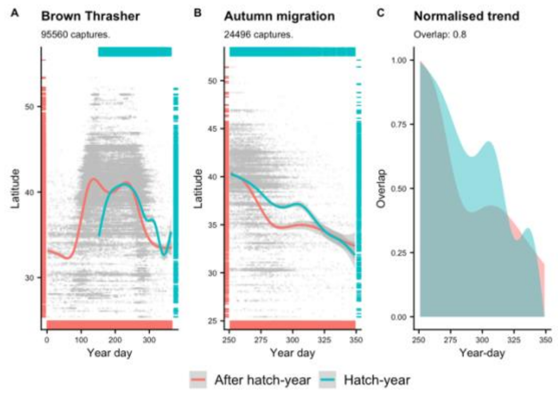


BOTW classification: Not classified

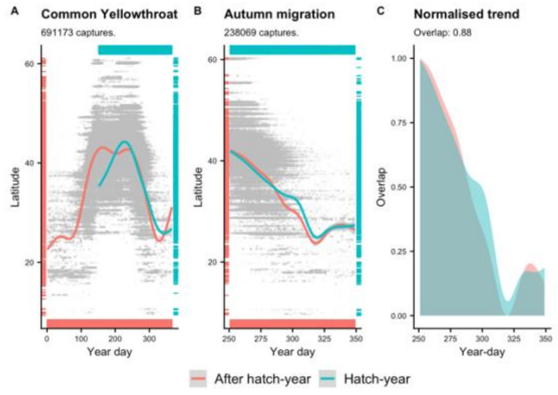


BOTW classification: Concurrent

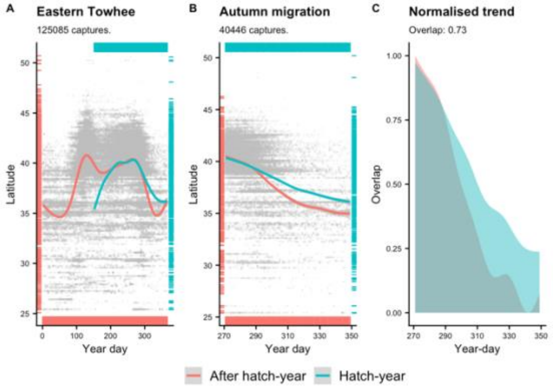


BOTW classification: Not classified

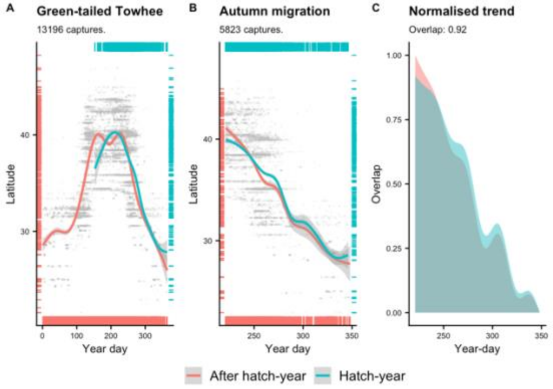


BOTW classification: Not classified

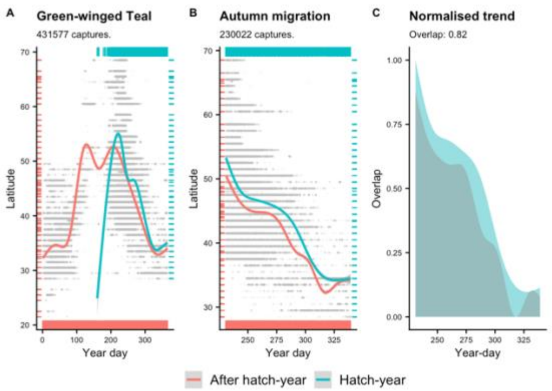


BOTW classification: Concurrent

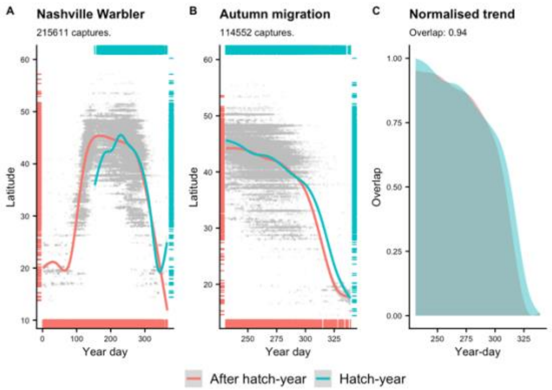


BOTW classification: Concurrent

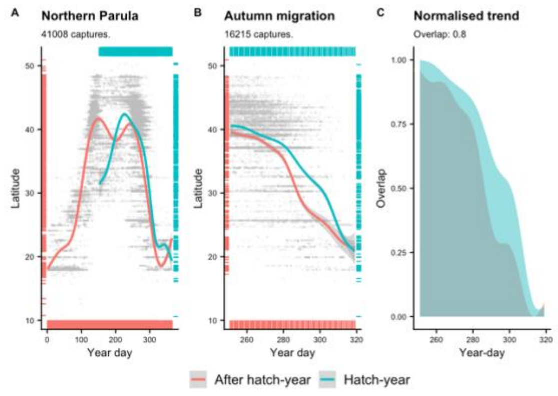


BOTW classification: Not classified

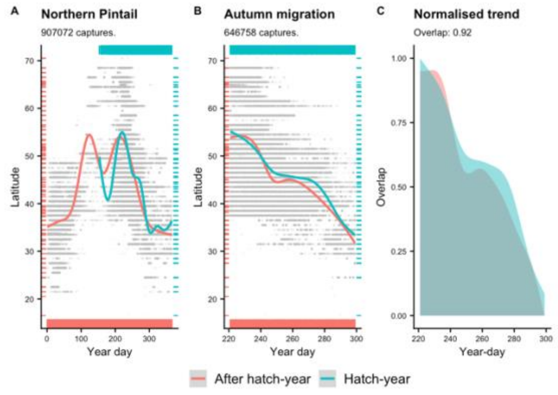


BOTW classification: Concurrent

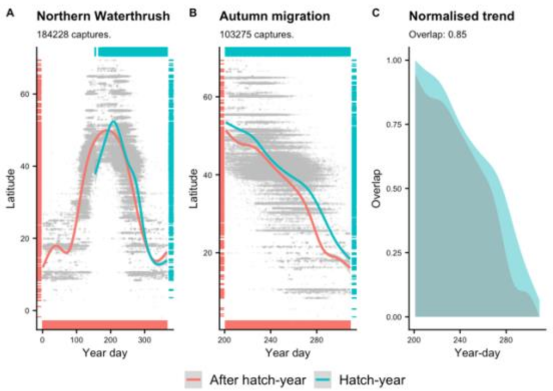


BOTW classification: Not classified

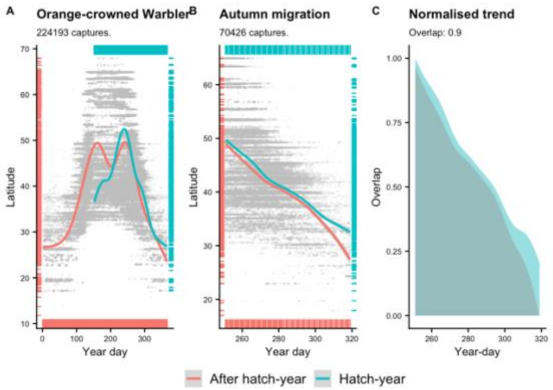


BOTW classification: Not classified

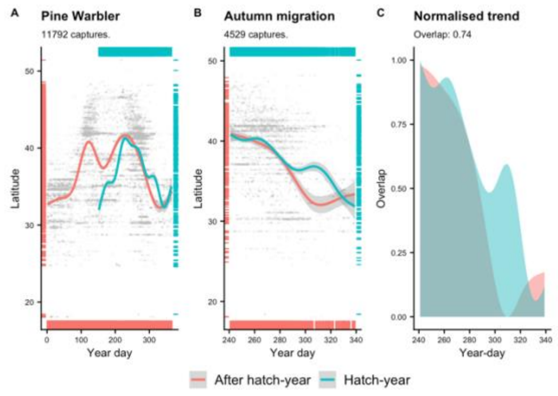


BOTW classification: Not classified

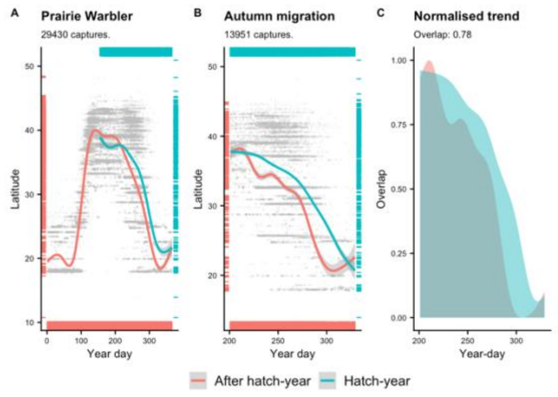


BOTW classification: Not classified

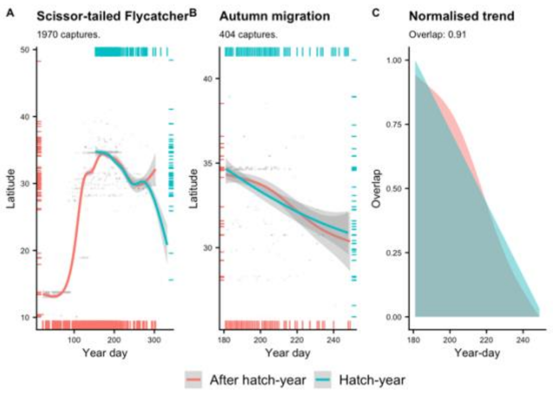


BOTW classification: Not classified

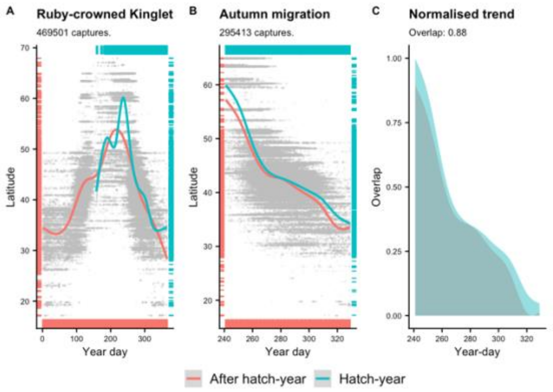


BOTW classification: Not classified

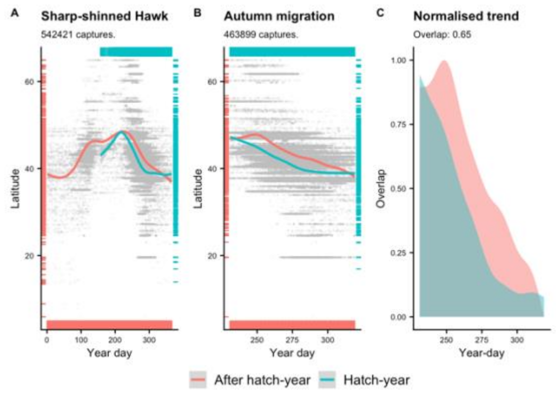


BOTW classification: Juveniles first

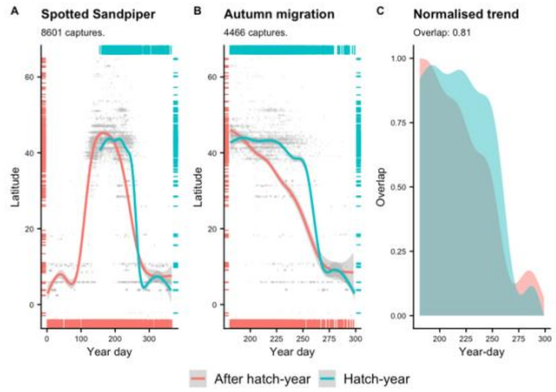


BOTW classification: Adults first

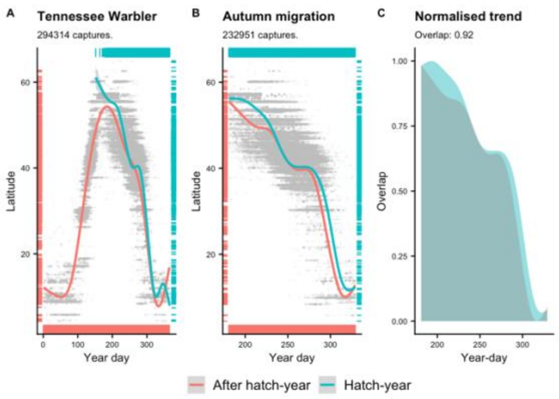


BOTW classification: Not classified

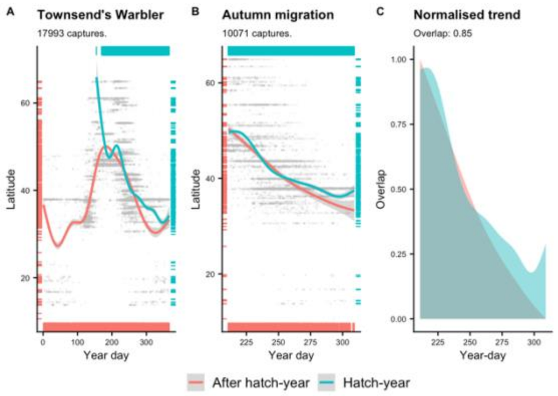


BOTW classification: Not classified

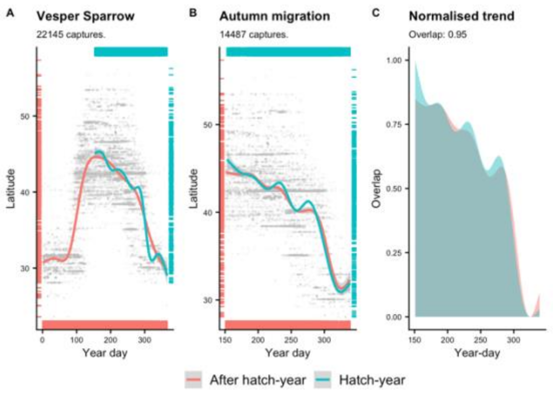


BOTW classification: Not classified

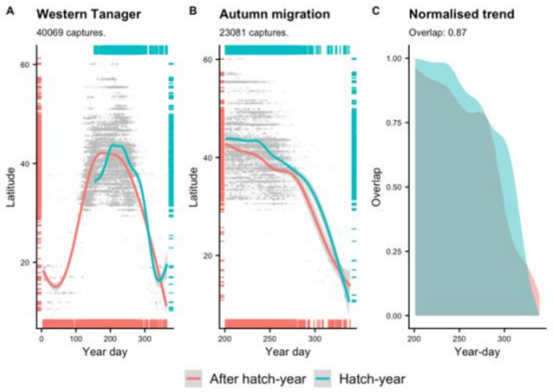


BOTW classification: Adults first

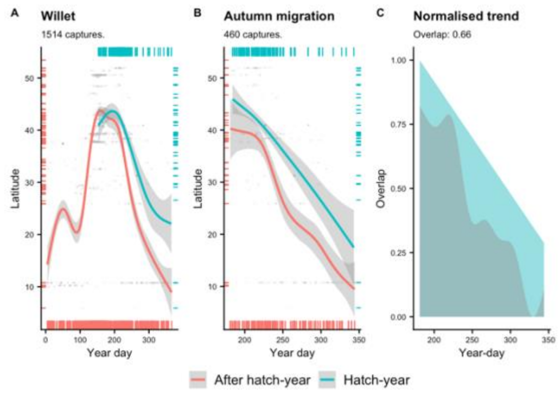


BOTW classification: Adults first

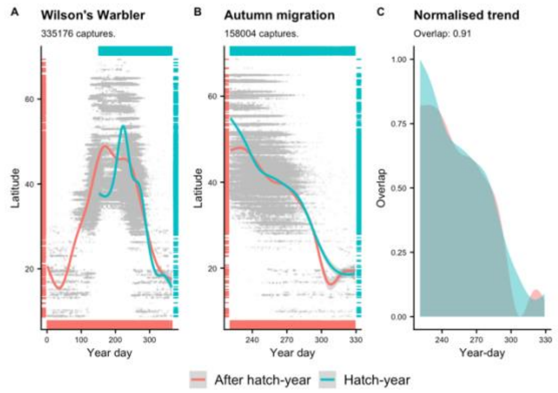


BOTW classification: Concurrent

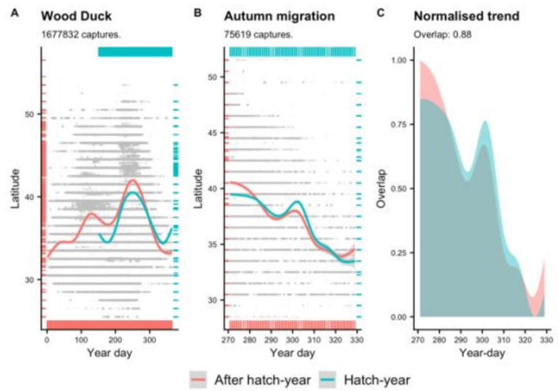


BOTW classification: Not classified

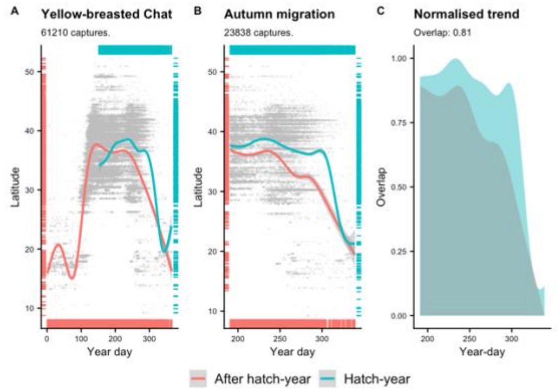


BOTW classification: Not classified

**Figure SM1.** Species timing plots for the 34 species included in PGLS model analysis whereby cohort-timing assessment was undertaken via analysis of USGS banding data. Start and end dates used for autumn migration period can be found in the full trait database dataset. Generalised Additive Model (GAM) model predicted average latitude of banding events across year-day for hatch-year and after hatch-year age cohorts across all USGS banding events 1960–2019 (plots labelled A). Time period was cropped to the autumn migration period where latitude is showing a clear negative trend (southward migration) (plots labelled B). Latitudes are normalised to a 0–1 scale and area under the curve overlap is calculated (plots labelled C).

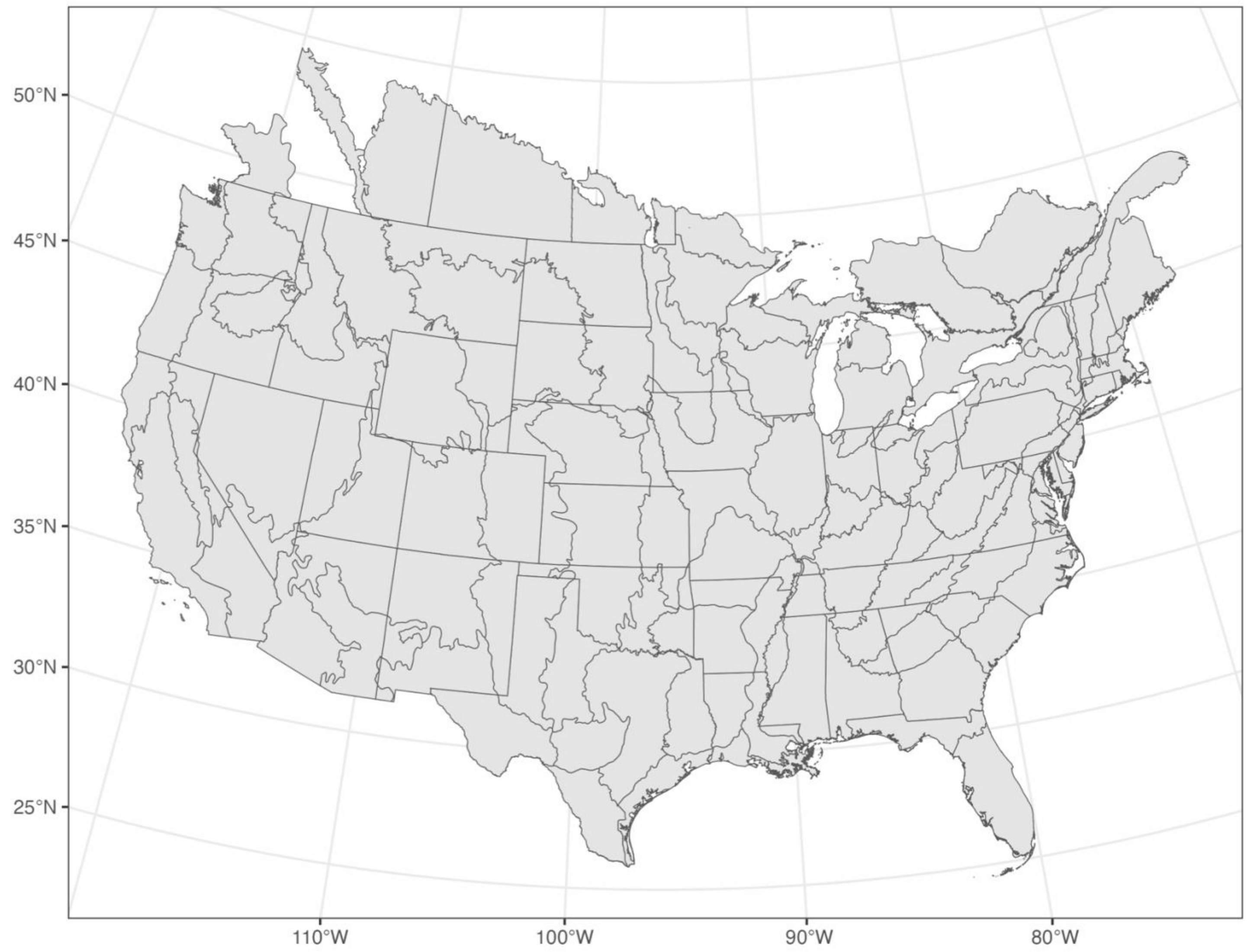

50°N
45°N
40°N
35°N
30°N
25°N
110°W
100°W
90°W
80°W

**Figure SM2.** Breeding Bird Survey and Christmas Bird Count analysis strata used to calculate annual centres of abundance 1970–2019. Strata refer to intersections between Bird Conservation Regions (BCRs) and states or provinces across North America.

# *SM2. PGLS analysis*

## ***SM2.1.*** *Breeding Bird Survey analysis*

### SM2.1.1 Flocking vs. Solo

Table SM1. Model set used for model averaging.

| X.Intercept. | Breeding_overlap_size | Breeding_rangesize | Combined_Migration_Timing | Diet_specialism_score | Flocks | GenFLS | Habitat_specialism_score | Mig_dist_numeric | Migration | Trend | df | logLik | AICc | delta | weight |
|---|---|---|---|---|---|---|---|---|---|---|---|---|---|---|---|
| 4038.35 | 314.96 | NA | + | NA | NA | NA | 510.75 | NA | NA | 1867.12 | 6 | -1222.09 | 2456.92 | 0 | 0.03 |
| 4501.13 | NA | NA | + | NA | NA | NA | 539.21 | NA | NA | 1588.84 | 5 | -1223.51 | 2457.54 | 0.63 | 0.02 |
| 3447.77 | 363.18 | NA | NA | NA | NA | NA | 421.55 | NA | NA | 2075.84 | 5 | -1223.64 | 2457.79 | 0.87 | 0.02 |
| 4266.28 | NA | 223.23 | + | NA | NA | NA | 551.57 | NA | NA | 1689.19 | 6 | -1222.65 | 2458.04 | 1.12 | 0.02 |
| 4021.36 | 320.7 | NA | + | NA | NA | -327.23 | 533.89 | NA | NA | 1905.69 | 7 | -1221.77 | 2458.53 | 1.61 | 0.01 |
| 4356.63 | 336.71 | NA | + | NA | + | NA | 540.2 | NA | NA | 1790.01 | 7 | -1221.8 | 2458.59 | 1.67 | 0.01 |
| 3509.34 | 316.55 | NA | NA | 235.42 | NA | NA | 365.33 | NA | NA | 2150.14 | 6 | -1222.99 | 2458.71 | 1.8 | 0.01 |
| 4007.28 | 292.76 | NA | + | 140 | NA | NA | 467.09 | NA | NA | 1935.23 | 7 | -1221.88 | 2458.74 | 1.83 | 0.01 |
| 3694.97 | NA | 278.35 | NA | NA | NA | NA | 470.83 | NA | NA | 1878.04 | 5 | -1224.16 | 2458.85 | 1.93 | 0.01 |
| 4410.11 | NA | NA | + | 200.1 | NA | NA | 473.95 | NA | NA | 1714.22 | 6 | -1223.07 | 2458.88 | 1.96 | 0.01 |

Table SM2. Model averaged coefficients. Full average used.

| Variable | Estimate | 2.50% | 97.50% | Significance | n_mod |
|---|---|---|---|---|---|
| Intercept | 4037.92 | 2644.56 | 5431.28 | Significant | 10 |

| | | | | | |
|---|---|---|---|---|---|
| Overlap range size | 201.23 | -229.55 | 632 | Non-significant | 10 |
| Migration timing (ref: day) | -758.21 | -2111.77 | 595.34 | Non-significant | 10 |
| Habitat specialism score | 492.91 | 73.24 | 912.58 | Significant | 10 |
| Absolute population trend | 1849.15 | 911.5 | 2786.81 | Significant | 10 |
| Total range size | 43.3 | -190.6 | 277.21 | Non-significant | 10 |
| Generation length | -27.11 | -324.17 | 269.95 | Non-significant | 10 |
| Flocking (ref: solo) | -37.67 | -471.66 | 396.33 | Non-significant | 10 |
| Diet specialism score | 42.11 | -214.44 | 298.66 | Non-significant | 10 |

### SM2.1.2 Age-structured flocking

Table SM3. Model set used for model averaging.

| X.Intercept. | Breeding_overlap_size | Breeding_rangesize | Combined_Migration_Timing | Diet_specialism_score | FlocksWithAdults | GenFLS | Habitat_specialism_score | Mig_dist_numeric | Migration | Trend | df | logLik | AICc | delta | weight |
|---|---|---|---|---|---|---|---|---|---|---|---|---|---|---|---|
| 3591.11 | NA | NA | NA | 385.46 | NA | NA | NA | NA | NA | 2832.43 | 4 | -793.93 | 1596.38 | 0 | 0.03 |
| 3757.15 | NA | -244.04 | NA | 446.76 | NA | NA | NA | NA | NA | 2615.04 | 5 | -792.82 | 1596.43 | 0.05 | 0.03 |
| 3899.79 | -272.48 | NA | NA | 467.15 | NA | NA | NA | NA | NA | 2619.91 | 5 | -793 | 1596.79 | 0.42 | 0.02 |
| 3473.14 | NA | NA | NA | NA | NA | NA | NA | NA | NA | 2741.08 | 3 | -795.26 | 1596.82 | 0.45 | 0.02 |
| 3547.45 | NA | NA | NA | NA | NA | NA | NA | 448.29 | NA | 2811.41 | 4 | -794.25 | 1597.02 | 0.65 | 0.02 |
| 3895.72 | NA | NA | NA | 363.21 | NA | NA | NA | NA | + | 2814.28 | 5 | -793.34 | 1597.48 | 1.1 | 0.02 |
| 3823.69 | NA | NA | NA | NA | NA | NA | NA | NA | + | 2726.59 | 4 | -794.53 | 1597.59 | 1.21 | 0.02 |
| 3624.89 | NA | NA | NA | 316.59 | NA | NA | NA | 330.99 | NA | 2868.04 | 5 | -793.41 | 1597.61 | 1.23 | 0.02 |
| 3626.04 | NA | NA | NA | NA | NA | NA | 306.59 | 521.51 | NA | 2609.49 | 5 | -793.41 | 1597.63 | 1.25 | 0.02 |
| 3587.92 | NA | -190.19 | NA | NA | NA | NA | NA | NA | NA | 2560.35 | 4 | -794.59 | 1597.7 | 1.32 | 0.02 |
| 3625.67 | NA | NA | NA | 361.76 | NA | NA | 192.93 | NA | NA | 2692.52 | 5 | -793.59 | 1597.98 | 1.6 | 0.01 |
| 3524.53 | NA | NA | NA | NA | NA | NA | 237.09 | NA | NA | 2576.05 | 4 | -794.75 | 1598.03 | 1.66 | 0.01 |
| 3656.22 | -175.84 | NA | NA | NA | NA | NA | NA | NA | NA | 2591.44 | 4 | -794.86 | 1598.24 | 1.87 | 0.01 |
| 3760.17 | NA | -215.19 | NA | 393.34 | NA | NA | NA | 221.93 | NA | 2664.62 | 6 | -792.59 | 1598.32 | 1.94 | 0.01 |

| | | | | | | | | | | | | | | | |
|---|---|---|---|---|---|---|---|---|---|---|---|---|---|---|---|
| 3521.58 | NA | NA | NA | 376.86 | NA | -266.73 | NA | NA | NA | 2818.67 | 5 | -793.78 | 1598.36 | 1.99 | 0.01 |

Table SM4. Model averaged coefficients. Full average used.

| Variable | Estimate | 2.50% | 97.50% | Significance | n_mod |
|---|---|---|---|---|---|
| Intercept | 3665.63 | 2538.11 | 4793.14 | Significant | 15 |
| Diet specialism score | 224.41 | -310.96 | 759.78 | Non-significant | 15 |
| Absolute population trend | 2708.72 | 1761.93 | 3655.52 | Significant | 15 |
| Total range size | -46.11 | -280.56 | 188.34 | Non-significant | 15 |
| Overlap range size | -32.09 | -252.38 | 188.19 | Non-significant | 15 |
| Migratory distance | 95.69 | -377.41 | 568.8 | Non-significant | 15 |
| Partial migrant (ref: complete) | -65.76 | -543.22 | 411.7 | Non-significant | 15 |
| Habitat specialism score | 39.15 | -223.83 | 302.13 | Non-significant | 15 |
| Generation length | -10.91 | -241.15 | 219.32 | Non-significant | 15 |

***SM2.2.*** *Christmas Bird Count analysis*

SM2.2.1 Flocking vs. Solo

Table SM5. Model set used for model averaging.

| X.Intercept. | Combined_Migration_Timing | Diet_specialism_score | Flocks | GenFLS | Habitat_specialism_score | Mig_dist_numeric | Migration | Nonbreeding_overlap_size | Nonbreeding_rangesize | Trend | df | logLik | AICc | delta | weight |
|---|---|---|---|---|---|---|---|---|---|---|---|---|---|---|---|
| 3058.7 | NA | NA | + | NA | 449.46 | NA | NA | NA | 550.96 | NA | 5 | -1303.81 | 2618.15 | 0 | 0.03 |
| 3204.28 | NA | NA | + | NA | 398.41 | 422.79 | NA | NA | 539.26 | NA | 6 | -1302.81 | 2618.36 | 0.21 | 0.02 |
| 2569.61 | + | NA | + | 419.11 | 373.74 | NA | NA | NA | 658.33 | NA | 7 | -1301.76 | 2618.5 | 0.36 | 0.02 |
| 2687.04 | + | NA | + | NA | 434.18 | NA | NA | NA | 624.77 | NA | 6 | -1303.1 | 2618.92 | 0.78 | 0.02 |
| 3350.35 | NA | NA | + | 285.4 | 375.77 | 448.89 | NA | NA | 530.17 | NA | 7 | -1302.01 | 2619.01 | 0.86 | 0.02 |
| 3184.44 | NA | NA | + | 261.77 | 431.6 | NA | NA | NA | 543.29 | NA | 6 | -1303.15 | 2619.03 | 0.88 | 0.02 |

| | | | | | | | | | | | | | | | |
|---|---|---|---|---|---|---|---|---|---|---|---|---|---|---|---|
| 3356.65 | NA | -288.94 | + | NA | 325 | 458.3 | NA | NA | 471.37 | NA | 7 | -1302.05 | 2619.07 | 0.93 | 0.02 |
| 3184.67 | NA | -266.57 | + | NA | 370.92 | NA | NA | NA | 482.51 | NA | 6 | -1303.2 | 2619.14 | 0.99 | 0.02 |
| 3170.12 | NA | -379.15 | + | NA | NA | 530.02 | NA | NA | 397.25 | NA | 6 | -1303.33 | 2619.38 | 1.24 | 0.01 |
| 2814.55 | + | NA | + | 404.88 | 344.41 | 329.12 | NA | NA | 625.64 | NA | 8 | -1301.2 | 2619.66 | 1.52 | 0.01 |
| 3008.29 | NA | NA | + | NA | 525.37 | 578.78 | NA | 273.84 | 440.54 | NA | 7 | -1302.44 | 2619.86 | 1.71 | 0.01 |
| 2369.27 | + | NA | + | 490.63 | NA | NA | NA | NA | 578.89 | NA | 6 | -1303.63 | 2619.98 | 1.84 | 0.01 |
| 2938.16 | + | NA | + | NA | 406.9 | 333.09 | NA | NA | 593.59 | NA | 7 | -1302.54 | 2620.06 | 1.91 | 0.01 |
| 2679.35 | NA | NA | + | NA | 397.12 | 585.42 | + | NA | 509.98 | NA | 7 | -1302.56 | 2620.11 | 1.96 | 0.01 |

Table SM6. Model averaged coefficients. Full average used.

| Variable | Estimate | 2.50% | 97.50% | Significance | n_mod |
|---|---|---|---|---|---|
| Intercept | 2995.97 | 1833.44 | 4158.51 | Significant | 14 |
| Flocking (ref: solo) | 1791.84 | 910.81 | 2672.88 | Significant | 14 |
| Habitat specialism score | 358.13 | -79.61 | 795.87 | Non-significant | 14 |
| Total range size | 543.54 | 197.19 | 889.89 | Significant | 14 |
| Migratory distance | 233.5 | -414.44 | 881.44 | Non-significant | 14 |
| Migration timing (ref: day) | 214.94 | -596.75 | 1026.63 | Non-significant | 14 |
| Generation length | 127.66 | -325.76 | 581.09 | Non-significant | 14 |
| Diet specialism score | -64.24 | -375.42 | 246.95 | Non-significant | 14 |
| Overlap range size | 13.62 | -170.95 | 198.2 | Non-significant | 14 |
| Partial migrant (ref: complete) | 27.4 | -423.4 | 478.2 | Non-significant | 14 |

SM2.2.2 Age-structured flocking

Table SM7. Model set used for model averaging.

| X.Intercept. | Combined_Migration_Timing | Diet_specialism_score | FlocksWithAdults | GenFLS | Habitat_specialism_score | Mig_dist_numeric | Migration | Nonbreeding_overlap_size | df | logLik | AICc | delta | weight |
|---|---|---|---|---|---|---|---|---|---|---|---|---|---|
| 4447.86 | + | NA | + | NA | NA | 741.63 | NA | NA | 6 | -858.66 | 1730.45 | 0 | 0.11 |
| 4424.67 | + | NA | + | 257.47 | NA | 719.67 | NA | NA | 7 | -858.18 | 1731.89 | 1.44 | 0.05 |
| 4585.76 | + | NA | + | NA | 167.31 | 683.9 | NA | NA | 7 | -858.34 | 1732.22 | 1.76 | 0.05 |

Table SM8. Model averaged coefficients. Full average used.

| Variable | Estimate | 2.50% | 97.50% | Significance | n_mod |
|---|---|---|---|---|---|
| Intercept | 4471.93 | 3450.77 | 5493.09 | Significant | 3 |
| Migration timing (ref: day) | -1276.23 | -2049.66 | -502.81 | Significant | 3 |
| Age-separated flocks (ref: solo) | 797.5 | -116.6 | 1711.59 | Non-significant | 3 |
| Mixed-age flocks (ref: solo) | 2660.87 | 1786.08 | 3535.67 | Significant | 3 |
| Migratory distance | 723.43 | 49.97 | 1396.89 | Significant | 3 |
| Generation length | 66 | -285.91 | 417.92 | Non-significant | 3 |
| Habitat specialism score | 36.42 | -207.15 | 279.98 | Non-significant | 3 |

## *SM3. Sensitivity analysis*

### ***SM3.1.*** *Breeding Bird Survey analysis*

#### SM3.1.1. Flocking as Solo – Small – Large

Table SM9. Model set used for model averaging.

| X.Intercept. | Breeding_overlap_size | Breeding_rangesize | Combined_Migration_Timing | Diet_specialism_score | Flock_size | GenFLS | Habitat_specialism_score | Mig_dist_numeric | Migration | Trend | df | logLik | AICc | delta | weight |
|---|---|---|---|---|---|---|---|---|---|---|---|---|---|---|---|
| 2893.69 | 865.85 | NA | NA | NA | NA | NA | NA | NA | NA | 1951.2 | 4 | -1051.22 | 2110.84 | 0 | 0.06 |
| 2994.51 | 809.16 | NA | NA | NA | NA | NA | 236.65 | NA | NA | 1931.08 | 5 | -1050.63 | 2111.86 | 1.03 | 0.03 |
| 2971.64 | 855.19 | NA | NA | NA | NA | 353.5 | NA | NA | NA | 1878.36 | 5 | -1050.85 | 2112.3 | 1.46 | 0.03 |

| | | | | | | | | | | | | | | | |
|---|---|---|---|---|---|---|---|---|---|---|---|---|---|---|---|
| 2733.89 | 850.07 | NA | NA | NA | + | NA | NA | NA | NA | 1902.4 | 6 | -1049.9 | 2112.66 | 1.83 | 0.02 |
| 2935.63 | 737.68 | 152.08 | NA | NA | NA | NA | NA | NA | NA | 1874.47 | 5 | -1051.09 | 2112.79 | 1.96 | 0.02 |

Table SM10. Model averaged coefficients. Full average used.

| Variable | Estimate | 2.50% | 97.50% | Significance | n_mod |
|---|---|---|---|---|---|
| Intercept | 2911.01 | 1720.05 | 4101.97 | Significant | 5 |
| Overlap range size | 833.09 | 332.4 | 1333.78 | Significant | 5 |
| Absolute population trend | 1917.77 | 990.59 | 2844.95 | Significant | 5 |
| Habitat specialism score | 49.6 | -226.24 | 325.44 | Non-significant | 5 |
| Generation length | 59.49 | -368.06 | 487.04 | Non-significant | 5 |
| Small flocks (ref: Solo) | 59.62 | NA | NA | NA | 5 |
| Large flocks (ref: Solo) | -66.72 | NA | NA | NA | 5 |
| Total range size | 20.01 | -224.81 | 264.84 | Non-significant | 5 |

### SM3.1.2. Binary Flocking variable – threshold of 5 (Beauchamp 2011 data)

Table SM11. Model set used for model averaging.

| X.Intercept. | Breeding_overlap_size | Combined_Migration_Timing | Diet_specialism_score | Flock_size | GenFLS | Habitat_specialism_score | Mig_dist_numeric | Migration | Trend | df | logLik | AICc | delta | weight |
|---|---|---|---|---|---|---|---|---|---|---|---|---|---|---|
| 3524.66 | NA | + | 470.97 | NA | -876.44 | NA | NA | + | 2481.06 | 7 | -713.95 | 1443.62 | 0 | 0.04 |
| 4238.78 | NA | + | 560.87 | NA | -740.81 | NA | -699.47 | NA | 2324.48 | 7 | -714.09 | 1443.91 | 0.29 | 0.03 |
| 4172.36 | NA | + | 574.21 | NA | NA | NA | -658.82 | NA | 2263.96 | 6 | -715.45 | 1444.17 | 0.55 | 0.03 |
| 3651.45 | NA | + | 393.23 | NA | -962.8 | 330.87 | NA | + | 2211.6 | 8 | -713.01 | 1444.27 | 0.65 | 0.03 |
| 3567.05 | NA | + | 478.54 | NA | NA | NA | NA | + | 2393.7 | 6 | -715.77 | 1444.81 | 1.19 | 0.02 |
| 3941.43 | NA | + | NA | NA | -1006.77 | 443.78 | NA | + | 1997.14 | 7 | -714.62 | 1444.96 | 1.34 | 0.02 |
| 3987.99 | 392.76 | + | NA | NA | -879.02 | 441.51 | NA | NA | 2232.12 | 7 | -714.76 | 1445.24 | 1.62 | 0.02 |
| 4040.27 | NA | + | 384.92 | NA | NA | NA | NA | NA | 2354.87 | 5 | -717.2 | 1445.3 | 1.68 | 0.02 |
| 4358.52 | NA | + | 490.48 | NA | -796.33 | 254.94 | -680.11 | NA | 2116.34 | 8 | -713.54 | 1445.32 | 1.7 | 0.02 |

| | | | | | | | | | | | | | | |
|---|---|---|---|---|---|---|---|---|---|---|---|---|---|---|
| 4093.41 | NA | + | 362.11 | NA | -675.58 | NA | NA | NA | 2415.17 | 6 | -716.13 | 1445.53 | 1.91 | 0.02 |
| 3811.96 | NA | + | 534.59 | NA | -836 | NA | -366.16 | + | 2409.11 | 8 | -713.68 | 1445.61 | 1.99 | 0.01 |

Table SM12. Model averaged coefficients. Full average used.

| Variable | Estimate | 2.50% | 97.50% | Significance | n_mod |
|---|---|---|---|---|---|
| Intercept | 3918.55 | 2397.88 | 5439.23 | Significant | 11 |
| Migration timing (ref: day) | -1703.31 | -3290.02 | -116.6 | Significant | 11 |
| Diet specialism score | 410.14 | -146.96 | 967.25 | Non-significant | 11 |
| Generation length | -622.8 | -1731.1 | 485.49 | Non-significant | 11 |
| Partial migrant (ref: complete) | 436.6 | -707.99 | 1581.19 | Non-significant | 11 |
| Absolute population trend | 2301.09 | 1372.26 | 3229.93 | Significant | 11 |
| Migratory distance | -236.76 | -1013.7 | 540.19 | Non-significant | 11 |
| Habitat specialism score | 119.29 | -329.46 | 568.04 | Non-significant | 11 |
| Overlap range size | 26.96 | -201.07 | 254.99 | Non-significant | 11 |

### SM3.1.3. Binary Flocking variable – threshold of 10 (Beauchamp 2011 data)

Table SM13. Model set used for model averaging.

| X.Intercept. | Breeding_overlap_size | Combined_Migration_Timing | Diet_specialism_score | Flock_size | GenFLS | Habitat_specialism_score | Mig_dist_numeric | Migration | Trend | df | logLik | AICc | delta | weight |
|---|---|---|---|---|---|---|---|---|---|---|---|---|---|---|
| 3524.66 | NA | + | 470.97 | NA | -876.44 | NA | NA | + | 2481.06 | 7 | -713.95 | 1443.62 | 0 | 0.04 |
| 4238.78 | NA | + | 560.87 | NA | -740.81 | NA | -699.47 | NA | 2324.48 | 7 | -714.09 | 1443.91 | 0.29 | 0.03 |
| 4172.36 | NA | + | 574.21 | NA | NA | NA | -658.82 | NA | 2263.96 | 6 | -715.45 | 1444.17 | 0.55 | 0.03 |
| 3651.45 | NA | + | 393.23 | NA | -962.8 | 330.87 | NA | + | 2211.6 | 8 | -713.01 | 1444.27 | 0.65 | 0.03 |
| 3567.05 | NA | + | 478.54 | NA | NA | NA | NA | + | 2393.7 | 6 | -715.77 | 1444.81 | 1.19 | 0.02 |
| 3941.43 | NA | + | NA | NA | -1006.77 | 443.78 | NA | + | 1997.14 | 7 | -714.62 | 1444.96 | 1.34 | 0.02 |
| 3987.99 | 392.76 | + | NA | NA | -879.02 | 441.51 | NA | NA | 2232.12 | 7 | -714.76 | 1445.24 | 1.62 | 0.02 |
| 4040.27 | NA | + | 384.92 | NA | NA | NA | NA | NA | 2354.87 | 5 | -717.2 | 1445.3 | 1.68 | 0.02 |
| 4358.52 | NA | + | 490.48 | NA | -796.33 | 254.94 | -680.11 | NA | 2116.34 | 8 | -713.54 | 1445.32 | 1.7 | 0.02 |

| | | | | | | | | | | | | | | |
|---|---|---|---|---|---|---|---|---|---|---|---|---|---|---|
| 4093.41 | NA | + | 362.11 | NA | -675.58 | NA | NA | NA | 2415.17 | 6 | -716.13 | 1445.53 | 1.91 | 0.02 |
| 3811.96 | NA | + | 534.59 | NA | -836 | NA | -366.16 | + | 2409.11 | 8 | -713.68 | 1445.61 | 1.99 | 0.01 |

Table SM14. Model averaged coefficients. Full average used.

| Variable | Estimate | 2.50% | 97.50% | Significance | n_mod |
|---|---|---|---|---|---|
| Intercept | 3918.55 | 2397.88 | 5439.23 | Significant | 11 |
| Migration timing (ref: day) | -1703.31 | -3290.02 | -116.6 | Significant | 11 |
| Diet specialism score | 410.14 | -146.96 | 967.25 | Non-significant | 11 |
| Generation length | -622.8 | -1731.1 | 485.49 | Non-significant | 11 |
| Partial migrant (ref: complete) | 436.6 | -707.99 | 1581.19 | Non-significant | 11 |
| Absolute population trend | 2301.09 | 1372.26 | 3229.93 | Significant | 11 |
| Migratory distance | -236.76 | -1013.7 | 540.19 | Non-significant | 11 |
| Habitat specialism score | 119.29 | -329.46 | 568.04 | Non-significant | 11 |
| Overlap range size | 26.96 | -201.07 | 254.99 | Non-significant | 11 |

## ***SM3.2.*** *Christmas Bird Count analysis*

### SM3.2.1. Flocking as Solo – Small – Large

Table SM15. Model set used for model averaging.

| X.Intercept. | Breeding_overlap_size | Breeding_rangesize | Combined_Migration_Timing | Diet_specialism_score | Flock_size | GenFLS | Habitat_specialism_score | Mig_dist_numeric | Migration | Trend | df | logLik | AICc | delta | weight |
|---|---|---|---|---|---|---|---|---|---|---|---|---|---|---|---|
| 3627.51 | NA | 807.93 | + | NA | + | 600.34 | NA | NA | + | NA | 8 | -1115.69 | 2248.88 | 0 | 0.03 |
| 2182.66 | NA | 754.95 | + | NA | + | 590.77 | NA | NA | NA | NA | 7 | -1116.98 | 2249.12 | 0.23 | 0.02 |
| 4216.98 | NA | 599.14 | NA | -421.93 | + | NA | NA | NA | + | NA | 7 | -1117.13 | 2249.42 | 0.53 | 0.02 |
| 4702.19 | NA | 696.38 | NA | -599.74 | + | 749.78 | 338.69 | NA | + | -879.92 | 10 | -1113.65 | 2249.63 | 0.75 | 0.02 |
| 4570.08 | NA | 649.82 | NA | -577.16 | + | 633.51 | NA | NA | + | -623.5 | 9 | -1114.91 | 2249.71 | 0.83 | 0.02 |

| | | | | | | | | | | | | | | | |
|---|---|---|---|---|---|---|---|---|---|---|---|---|---|---|---|
| 2794.4 | NA | 537.14 | NA | -424.83 | + | NA | NA | NA | NA | NA | 6 | -1118.43 | 2249.72 | 0.84 | 0.02 |
| 3342.49 | NA | 640.41 | NA | -602.85 | + | 734.17 | 375.18 | NA | NA | -889.99 | 9 | -1114.93 | 2249.76 | 0.88 | 0.02 |
| 2365.49 | NA | 770.51 | + | NA | + | 559.43 | 232.46 | NA | NA | NA | 8 | -1116.27 | 2250.04 | 1.15 | 0.01 |
| 4422.78 | NA | 611.51 | NA | -351.74 | + | 316.24 | NA | NA | + | NA | 8 | -1116.27 | 2250.05 | 1.16 | 0.01 |
| 3684.63 | NA | 817.69 | + | NA | + | 572.57 | 201.03 | NA | + | NA | 9 | -1115.15 | 2250.2 | 1.32 | 0.01 |
| 3788.43 | NA | 749.52 | + | -204.71 | + | 489.28 | NA | NA | + | NA | 9 | -1115.23 | 2250.36 | 1.48 | 0.01 |
| 3033.53 | NA | 581.61 | NA | -577.92 | + | 602.04 | NA | NA | NA | -603.86 | 8 | -1116.46 | 2250.41 | 1.53 | 0.01 |
| 3605.86 | 229.64 | 663.06 | + | NA | + | 578.01 | NA | NA | + | NA | 9 | -1115.26 | 2250.42 | 1.53 | 0.01 |
| 2932.05 | NA | 546.32 | NA | -359.39 | + | 295.34 | NA | NA | NA | NA | 7 | -1117.7 | 2250.56 | 1.68 | 0.01 |
| 2970.09 | NA | 544.3 | NA | -421.66 | + | NA | NA | 364.8 | NA | NA | 7 | -1117.71 | 2250.57 | 1.69 | 0.01 |
| 2335.24 | NA | 697.05 | + | -202.12 | + | 481.07 | NA | NA | NA | NA | 8 | -1116.54 | 2250.59 | 1.71 | 0.01 |
| 4147.15 | 267.55 | 442.6 | NA | -402.51 | + | NA | NA | NA | + | NA | 8 | -1116.56 | 2250.61 | 1.73 | 0.01 |
| 4495.65 | 295.75 | 478.92 | NA | -573.27 | + | 643.83 | NA | NA | + | -665.36 | 10 | -1114.18 | 2250.7 | 1.82 | 0.01 |

Table SM16. Model averaged coefficients. Full average used.

| Variable | Estimate | 2.50% | 97.50% | Significance | n_mod |
|---|---|---|---|---|---|
| Intercept | 3511.31 | 1199.18 | 5823.44 | Significant | 18 |
| Total range size | 658.82 | 264.01 | 1053.63 | Significant | 18 |
| Migration timing (ref: day) | 394.85 | -726.45 | 1516.16 | Non-significant | 18 |
| Small flocks (ref: Solo) | 1513.31 | 539.07 | 2487.55 | Significant | 18 |
| Large flocks (ref: Solo) | 2721.56 | -262.87 | 5705.99 | Non-significant | 18 |
| Generation length | 448.75 | -259.06 | 1156.55 | Non-significant | 18 |
| Partial migrant (ref: complete) | -878.81 | -2952.13 | 1194.52 | Non-significant | 18 |

| | | | | | |
|---|---|---|---|---|---|
| Diet specialism score | -303.05 | -887.14 | 281.05 | Non-significant | 18 |
| Habitat specialism score | 67.81 | -255.51 | 391.12 | Non-significant | 18 |
| Absolute population trend | -204.51 | -993.45 | 584.42 | Non-significant | 18 |
| Overlap range size | 32.32 | -214.53 | 279.17 | Non-significant | 18 |
| Migratory distance | 15.01 | -174.18 | 204.2 | Non-significant | 18 |

SM3.2.2. Binary Flocking variable – threshold of 5 (Beauchamp 2011 data)

Table SM17. Model set used for model averaging.

| X.Intercept. | Breeding_overlap_size | Breeding_rangesize | Combined_Migration_Timing | Diet_specialism_score | Flock_size | GenFLS | Habitat_specialism_score | Mig_dist_numeric | Migration | Trend | df | logLik | AICc | delta | weight |
|---|---|---|---|---|---|---|---|---|---|---|---|---|---|---|---|
| 4235.45 | NA | NA | NA | NA | + | 1638.41 | NA | 658.61 | NA | -1022.47 | 6 | -776.24 | 1565.75 | 0 | 0.07 |
| 3849.03 | NA | NA | NA | NA | + | 1542.56 | NA | NA | NA | -974.38 | 5 | -777.87 | 1566.64 | 0.89 | 0.05 |
| 4434.26 | NA | NA | NA | -374.82 | + | 1550.03 | NA | 723.54 | NA | -1178.43 | 7 | -775.56 | 1566.85 | 1.1 | 0.04 |
| 3920.51 | NA | NA | NA | NA | + | 1577.91 | -265.59 | 737.43 | NA | -854.04 | 7 | -775.83 | 1567.39 | 1.65 | 0.03 |

Table SM18. Model averaged coefficients. Full average used.

| Variable | Estimate | 2.50% | 97.50% | Significance | n_mod |
|---|---|---|---|---|---|
| Intercept | 4133.43 | 3266.07 | 5000.78 | Significant | 4 |
| Flocking (ref: Solo/Small) | 1373.27 | 425.82 | 2320.73 | Significant | 4 |
| Generation length | 1586.11 | 845.7 | 2326.53 | Significant | 4 |
| Migratory distance | 526.96 | -353.41 | 1407.33 | Non-significant | 4 |
| Absolute population trend | -1016.92 | -1721.83 | -312.01 | Significant | 4 |
| Diet specialism score | -81.4 | -494.47 | 331.67 | Non-significant | 4 |
| Habitat specialism score | -43.88 | -360.14 | 272.37 | Non-significant | 4 |

SM3.2.3. Binary Flocking variable – threshold of 10 (Beauchamp 2011 data)

Table SM19. Model set used for model averaging.

| X.Intercept. | Breeding_overlap_size | Breeding_rangesize | Combined_Migration_Timing | Diet_specialism_score | Flock_size | GenFLS | Habitat_specialism_score | Mig_dist_numeric | Migration | Trend | df | logLik | AICc | delta | weight |
|---|---|---|---|---|---|---|---|---|---|---|---|---|---|---|---|
| 3930.7 | NA | NA | NA | NA | + | 1428.52 | NA | NA | NA | -730.31 | 5 | -776.02 | 1562.94 | 0 | 0.06 |
| 4293.08 | NA | NA | NA | NA | + | 1328.42 | NA | 617.97 | NA | -645.22 | 6 | -774.91 | 1563.1 | 0.16 | 0.05 |
| 4417.82 | NA | NA | NA | -308.39 | + | 1386.87 | NA | 605.52 | NA | -876.39 | 7 | -774.34 | 1564.4 | 1.46 | 0.03 |
| 3412.58 | NA | NA | NA | NA | + | 1321.54 | NA | 927.01 | + | -640.89 | 7 | -774.38 | 1564.48 | 1.54 | 0.03 |
| 4031.48 | 298.83 | NA | NA | NA | + | 1381.94 | NA | 626.33 | NA | -723.59 | 7 | -774.41 | 1564.55 | 1.61 | 0.02 |
| 4077.65 | NA | NA | NA | -323.85 | + | 1358.39 | NA | NA | NA | -889.22 | 6 | -775.72 | 1564.71 | 1.77 | 0.02 |
| 5322.37 | NA | NA | + | -720.33 | + | 1200.44 | NA | 828.66 | NA | -1131.13 | 8 | -773.25 | 1564.76 | 1.82 | 0.02 |

Table SM20. Model averaged coefficients. Full average used.

| Variable | Estimate | 2.50% | 97.50% | Significance | n_mod |
|---|---|---|---|---|---|
| Intercept | 4172.07 | 2801.39 | 5542.76 | Significant | 7 |
| Flocking (ref: Solo/Small) | 1625.77 | 634.5 | 2617.04 | Significant | 7 |
| Generation length | 1355 | 611.87 | 2098.12 | Significant | 7 |
| Absolute population trend | -772.54 | -1494.14 | -50.93 | Significant | 7 |
| Migratory distance | 461.33 | -482.87 | 1405.54 | Non-significant | 7 |
| Diet specialism score | -138.57 | -733.88 | 456.75 | Non-significant | 7 |
| Partial migrant (ref: complete) | 121.37 | -870.44 | 1113.17 | Non-significant | 7 |
| Overlap range size | 32.37 | -241.29 | 306.03 | Non-significant | 7 |
| Migration timing (ref: day) | -134.24 | -1083 | 814.52 | Non-significant | 7 |

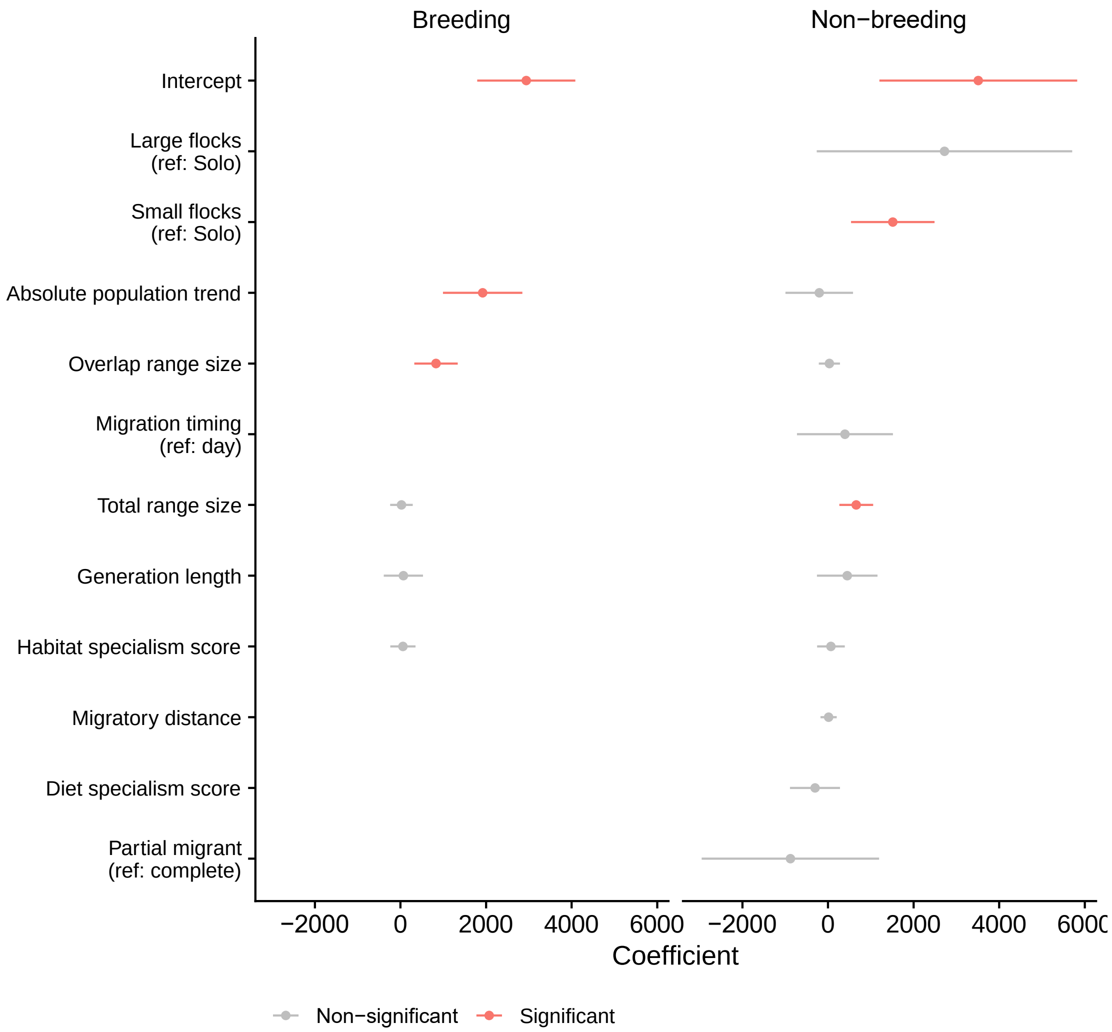


Figure SM3. Phylogenetic Generalised Least Squares (PGLS) model coefficients for predictors of annual shift rates of Breeding Bird Survey (left) and Christmas Bird Count (right) centre of abundance (in metres) 1970–2019. Models assessed migratory flocking as solo, small (<10 individuals), and large (>10 individuals) for 105 species. Red points indicate significant results inferred from credible 95th percentiles (error bars) that exclude zero.

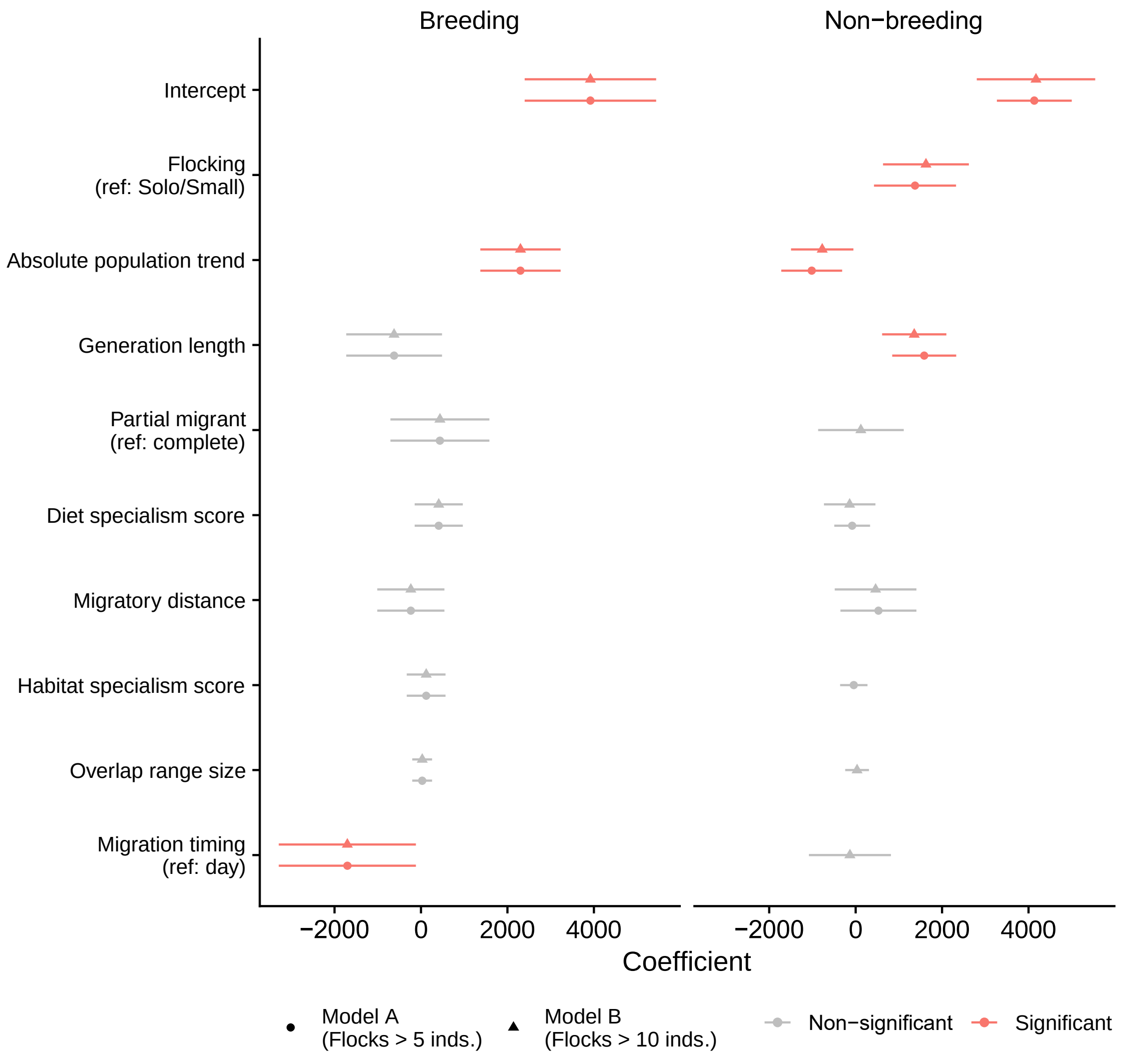


Figure SM4. Phylogenetic Generalised Least Squares (PGLS) model coefficients for predictors of annual shift rates of Breeding Bird Survey (left) and Christmas Bird Count (right) centre of abundance (in metres) 1970–2019 for 73 species. Model A assessed migratory flocking as a binary flocking vs. solo migrants with flocking species defined as those with maximum flock

size while travelling of >5 from Beachamp (2011) and Model B with a threshold of >10 individuals. Red points indicate significant results inferred from credible 95th percentiles (error bars) that exclude zero.

## *SM4. Birds of the World – Reference list*

Table SM21. Birds of the World references for each species used in PGLS analysis.

| AOU | Genus | Species | BOTW_Ref |
|---|---|---|---|
| 20 | Podiceps | grisegena | Stout, B. E. & G. L. Nuechterlein (2020). Red-necked Grebe (Podiceps grisegena), version 1.0. In Birds of the World (S. M. Billerman, Editor). Cornell Lab of Ornithology, Ithaca, NY, USA. |
| 40 | Podiceps | nigricollis | Cullen, S. A., J. R. Jehl Jr., & G. L. Nuechterlein (2020). Eared Grebe (Podiceps nigricollis), version 1.0. In Birds of the World (S. M. Billerman, Editor). Cornell Lab of Ornithology, Ithaca, NY, USA. |
| 470 | Larus | marinus | Good, T. P. (2020). Great Black-backed Gull (*Larus marinus*), version 1.0. In Birds of the World (S. M. Billerman, Editor). Cornell Lab of Ornithology, Ithaca, NY, USA. |
| 580 | Leucophaeus | atricilla | Burger, J. (2020). Laughing Gull (*Leucophaeus atricilla*), version 1.0. In Birds of the World (P. G. Rodewald, Editor). Cornell Lab of Ornithology, Ithaca, NY, USA. |
| 590 | Leucophaeus | pipixcan | Burger, J. & M. Gochfeld (2020). Franklin's Gull (Leucophaeus pipixcan), version 1.0. In Birds of the World (A. F. Poole, Editor). Cornell Lab of Ornithology, Ithaca, NY, USA. |
| 640 | Hydroprogne | caspia | Cuthbert, F. J. & L. R. Wires (2020). Caspian Tern (Hydroprogne caspia), version 1.0. In Birds of the World (S. M. Billerman, Editor). Cornell Lab of Ornithology, Ithaca, NY, USA. |
| 650 | Thalasseus | maximus | Buckley, P. A., F. G. Buckley, & S. G. Mlodinow (2021). Royal Tern (Thalasseus maximus), version 1.1. In Birds of the World (S. M. Billerman, Editor). Cornell Lab of Ornithology, Ithaca, NY, USA. |
| 690 | Sterna | forsteri | McNicholl, M. K., P. E. Lowther, & J. A. Hall (2020). Forster's Tern (Sterna forsteri), version 1.0. In Birds of the World (A. F. Poole & F. B. Gill, Editors). Cornell Lab of Ornithology, Ithaca, NY, USA. |
| 800 | Rynchops | niger | Gochfeld, M., J. Burger, & K. L. Lefevre (2020). Black Skimmer (Rynchops niger), version 1.0. In Birds of the World (S. M. Billerman, Editor). Cornell Lab of Ornithology, Ithaca, NY, USA. |
| 1230 | Urile | pelagicus | Hobson, K. A. (2021). Pelagic Cormorant (*Urile pelagicus*), version 1.1. In Birds of the World (Editor not available). Cornell Lab of Ornithology, Ithaca, NY, USA. |
| 1250 | Pelecanus | erythrorhynchos | Knopf, F. L. & R. M. Evans (2020). American White Pelican (Pelecanus erythrorhynchos), version 1.0. In Birds of the World (A. F. Poole, Editor). Cornell Lab of Ornithology, Ithaca, NY, USA. |
| 1260 | Pelecanus | occidentalis | Shields, M. (2020). Brown Pelican (*Pelecanus occidentalis*), version 1.0. In Birds of the World (A. F. Poole, Editor). Cornell Lab of Ornithology, Ithaca, NY, USA. |

| | | | |
|---|---|---|---|
| 1290 | Mergus | merganser | Pearce, J., M. L. Mallory, & K. Metz (2020). Common Merganser (Mergus merganser), version 1.0. In Birds of the World (S. M. Billerman, Editor). Cornell Lab of Ornithology, Ithaca, NY, USA. |
| 1310 | Lophodytes | cucullatus | Dugger, B. D., K. M. Dugger, & L. H. Fredrickson (2020). Hooded Merganser (Lophodytes cucullatus), version 1.0. In Birds of the World (A. F. Poole, Editor). Cornell Lab of Ornithology, Ithaca, NY, USA. |
| 1350 | Mareca | strepera | Leschack, C. R., S. K. McKnight, & G. R. Hepp (2020). Gadwall (Mareca strepera), version 1.0. In Birds of the World (S. M. Billerman, Editor). Cornell Lab of Ornithology, Ithaca, NY, USA. |
| 1370 | Mareca | americana | Mini, A. E., E. R. Harrington, E. Rucker, B. D. Dugger, & T. B. Mowbray (2020). American Wigeon (Mareca americana), version 1.0. In Birds of the World (A. F. Poole, Editor). Cornell Lab of Ornithology, Ithaca, NY, USA. |
| 1390 | Anas | crecca | Johnson, K., C. Carboneras, D. A. Christie, & G. M. Kirwan (2020). Green-winged Teal (Anas crecca), version 1.0. In Birds of the World (S. M. Billerman, Editor). Cornell Lab of Ornithology, Ithaca, NY, USA. |
| 1400 | Spatula | discors | Rohwer, F. C., W. P. Johnson, & E. R. Loos (2020). Blue-winged Teal (Spatula discors), version 1.0. In Birds of the World (A. F. Poole & F. B. Gill, Editors). Cornell Lab of Ornithology, Ithaca, NY, USA. |
| 1410 | Spatula | cyanoptera | Gammonley, J. H. (2020). Cinnamon Teal (*Spatula cyanoptera*), version 1.0. In Birds of the World (A. F. Poole, Editor). Cornell Lab of Ornithology, Ithaca, NY, USA. |
| 1430 | Anas | acuta | Clark, R. G., J. P. Fleskes, K. L. Guyn, D. A. Haukos, J. E. Austin, & M. R. Miller (2020). Northern Pintail (Anas acuta), version 1.0. In Birds of the World (S. M. Billerman, Editor). Cornell Lab of Ornithology, Ithaca, NY, USA. |
| 1440 | Aix | sponsa | Hepp, G. R. & F. C. Bellrose (2020). Wood Duck (Aix sponsa), version 1.0. In Birds of the World (A. F. Poole, Editor). Cornell Lab of Ornithology, Ithaca, NY, USA. |
| 1490 | Aythya | affinis | Anteau, M. J., J. DeVink, D. N. Koons, J. E. Austin, C. M. Custer, & A. D. Afton (2020). Lesser Scaup (Aythya affinis), version 1.0. In Birds of the World (A. F. Poole, Editor). Cornell Lab of Ornithology, Ithaca, NY, USA. |
| 1500 | Aythya | collaris | Roy, C. L., C. M. Herwig, W. L. Hohman, & R. T. Eberhardt (2020). Ring-necked Duck (Aythya collaris), version 1.0. In Birds of the World (A. F. Poole, Editor). Cornell Lab of Ornithology, Ithaca, NY, USA. |
| 1510 | Bucephala | clangula | Eadie, J. M., M. L. Mallory, & H. G. Lumsden (2020). Common Goldeneye (Bucephala clangula), version 1.0. In Birds of the World (S. M. Billerman, Editor). Cornell Lab of Ornithology, Ithaca, NY, USA. |
| 1530 | Bucephala | albeola | Gauthier, G. (2020). Bufflehead (*Bucephala albeola*), version 1.0. In Birds of the World (A. F. Poole, Editor). Cornell Lab of Ornithology, Ithaca, NY, USA. |
| 1770 | Dendrocygna | autumnalis | James, J. D. & J. E. Thompson (2020). Black-bellied Whistling-Duck (Dendrocygna autumnalis), version 1.0. In Birds of the World (A. F. Poole & F. B. Gill, Editors). Cornell Lab of Ornithology, Ithaca, NY, USA. |
| 1780 | Dendrocygna | bicolor | Hohman, W. L. & S. A. Lee (2020). Fulvous Whistling-Duck (Dendrocygna bicolor), version 1.0. In Birds of the World (S. M. Billerman, Editor). Cornell Lab of Ornithology, Ithaca, NY, USA. |
| 1860 | Plegadis | falcinellus | Davis Jr., W. E. & J. C. Kricher (2020). Glossy Ibis (Plegadis falcinellus), version 1.0. In Birds of the World (S. M. Billerman, Editor). Cornell Lab of Ornithology, Ithaca, NY, USA. |
| 1900 | Botaurus | lentiginosus | Lowther, P. E., A. F. Poole, J. P. Gibbs, S. M. Melvin, & F. A. Reid (2020). American Bittern (Botaurus lentiginosus), version 1.0. In Birds of the World (A. F. Poole, Editor). Cornell Lab of Ornithology, Ithaca, NY, USA. |

| | | | |
|---|---|---|---|
| 1960 | Ardea | alba | McCrimmon Jr., D. A., J. C. Ogden, G. T. Bancroft, A. Martínez-Vilalta, A. Motis, G. M. Kirwan, & P. F. D. Boesman (2020). Great Egret (Ardea alba), version 1.0. In Birds of the World (S. M. Billerman, Editor). Cornell Lab of Ornithology, Ithaca, NY, USA. |
| 2000 | Egretta | caerulea | Rodgers Jr., J. A. & H. T. Smith (2020). Little Blue Heron (Egretta caerulea), version 1.0. In Birds of the World (A. F. Poole, Editor). Cornell Lab of Ornithology, Ithaca, NY, USA. |
| 2001 | Bubulcus | ibis | Telfair II, R. C. (2020). Cattle Egret (*Bubulcus ibis*), version 1.0. In Birds of the World (S. M. Billerman, Editor). Cornell Lab of Ornithology, Ithaca, NY, USA. |
| 2010 | Butorides | virescens | Davis Jr., W. E. & J. A. Kushlan (2020). Green Heron (Butorides virescens), version 1.0. In Birds of the World (A. F. Poole & F. B. Gill, Editors). Cornell Lab of Ornithology, Ithaca, NY, USA. |
| 2060 | Antigone | canadensis | Gerber, B. D., J. F. Dwyer, S. A. Nesbitt, R. C. Drewien, C. D. Littlefield, T. C. Tacha, & P. A. Vohs (2020). S&hill Crane (Antigone canadensis), version 1.0. In Birds of the World (A. F. Poole, Editor). Cornell Lab of Ornithology, Ithaca, NY, USA. |
| 2080 | Rallus | elegans | Pickens, B. A. & B. Meanley (2020). King Rail (Rallus elegans), version 1.0. In Birds of the World (P. G. Rodewald, Editor). Cornell Lab of Ornithology, Ithaca, NY, USA. |
| 2120 | Rallus | limicola | Conway, C. J. (2020). Virginia Rail (Rallus limicola), version 1.0. In Birds of the World (A. F. Poole & F. B. Gill, Editors). Cornell Lab of Ornithology, Ithaca, NY, USA. |
| 2140 | Porzana | carolina | Melvin, S. M. & J. P. Gibbs (2020). Sora (Porzana carolina), version 1.0. In Birds of the World (A. F. Poole, Editor). Cornell Lab of Ornithology, Ithaca, NY, USA. |
| 2210 | Fulica | americana | Brisbin Jr., I. L. & T. B. Mowbray (2020). American Coot (Fulica americana), version 1.0. In Birds of the World (A. F. Poole & F. B. Gill, Editors). Cornell Lab of Ornithology, Ithaca, NY, USA. |
| 2250 | Recurvirostra | americana | Ackerman, J. T., C. A. Hartman, M. P. Herzog, J. Y. Takekawa, J. A. Robinson, L. W. Oring, J. P. Skorupa, & R. Boettcher (2020). American Avocet (Recurvirostra americana), version 1.0. In Birds of the World (A. F. Poole, Editor). Cornell Lab of Ornithology, Ithaca, NY, USA. |
| 2260 | Himantopus | mexicanus | Robinson, J. A., J. M. Reed, J. P. Skorupa, & L. W. Oring (2020). Black-necked Stilt (Himantopus mexicanus), version 1.0. In Birds of the World (A. F. Poole & F. B. Gill, Editors). Cornell Lab of Ornithology, Ithaca, NY, USA. |
| 2280 | Scolopax | minor | McAuley, D. G., D. M. Keppie, & R. M. Whiting Jr. (2020). American Woodcock (Scolopax minor), version 1.0. In Birds of the World (A. F. Poole, Editor). Cornell Lab of Ornithology, Ithaca, NY, USA. |
| 2490 | Limosa | fedoa | Gratto-Trevor, C. L. (2020). Marbled Godwit (Limosa fedoa), version 1.0. In Birds of the World (A. F. Poole & F. B. Gill, Editors). Cornell Lab of Ornithology, Ithaca, NY, USA. |
| 2540 | Tringa | melanoleuca | Elphick, C. S. & T. L. Tibbitts (2020). Greater Yellowlegs (Tringa melanoleuca), version 1.0. In Birds of the World (A. F. Poole & F. B. Gill, Editors). Cornell Lab of Ornithology, Ithaca, NY, USA. |
| 2550 | Tringa | flavipes | Tibbitts, T. L. & W. Moskoff (2020). Lesser Yellowlegs (Tringa flavipes), version 1.0. In Birds of the World (A. F. Poole, Editor). Cornell Lab of Ornithology, Ithaca, NY, USA. |
| 2560 | Tringa | solitaria | Moskoff, W. (2020). Solitary S&piper (Tringa solitaria), version 1.0. In Birds of the World (A. F. Poole, Editor). Cornell Lab of Ornithology, Ithaca, NY, USA. |

| | | | |
|---|---|---|---|
| 2580 | Tringa | semipalmata | Lowther, P. E., H. D. Douglas III, & C. L. Gratto-Trevor (2020). Willet (Tringa semipalmata), version 1.0. In Birds of the World (A. F. Poole & F. B. Gill, Editors). Cornell Lab of Ornithology, Ithaca, NY, USA. |
| 2630 | Actitis | macularius | Reed, J. M., L. W. Oring, & E. M. Gray (2020). Spotted S&piper (Actitis macularius), version 1.0. In Birds of the World (A. F. Poole, Editor). Cornell Lab of Ornithology, Ithaca, NY, USA. |
| 2640 | Numenius | americanus | Dugger, B. D. & K. M. Dugger (2020). Long-billed Curlew (Numenius americanus), version 1.0. In Birds of the World (A. F. Poole & F. B. Gill, Editors). Cornell Lab of Ornithology, Ithaca, NY, USA. |
| 2730 | Charadrius | vociferus | Jackson, B. J. & J. A. Jackson (2020). Killdeer (Charadrius vociferus), version 1.0. In Birds of the World (A. F. Poole & F. B. Gill, Editors). Cornell Lab of Ornithology, Ithaca, NY, USA. |
| 3120 | Patagioenas | fasciata | Keppie, D. M. & C. E. Braun (2020). B&-tailed Pigeon (Patagioenas fasciata), version 1.0. In Birds of the World (A. F. Poole & F. B. Gill, Editors). Cornell Lab of Ornithology, Ithaca, NY, USA. |
| 3160 | Zenaida | macroura | Otis, D. L., J. H. Schulz, D. Miller, R. E. Mirarchi, & T. S. Baskett (2020). Mourning Dove (Zenaida macroura), version 1.0. In Birds of the World (A. F. Poole, Editor). Cornell Lab of Ornithology, Ithaca, NY, USA. |
| 3190 | Zenaida | asiatica | Schwertner, T. W., H. A. Mathewson, J. A. Roberson, & G. L. Waggerman (2020). White-winged Dove (Zenaida asiatica), version 1.0. In Birds of the World (A. F. Poole & F. B. Gill, Editors). Cornell Lab of Ornithology, Ithaca, NY, USA. |
| 3250 | Cathartes | aura | Kirk, D. A. & M. J. Mossman (2020). Turkey Vulture (Cathartes aura), version 1.0. In Birds of the World (A. F. Poole & F. B. Gill, Editors). Cornell Lab of Ornithology, Ithaca, NY, USA. |
| 3260 | Coragyps | atratus | Buckley, N. J., B. M. Kluever, R. Driver, & S. A. Rush (2022). Black Vulture (Coragyps atratus), version 2.0. In Birds of the World (P. G. Rodewald & B. K. Keeney, Editors). Cornell Lab of Ornithology, Ithaca, NY, USA. |
| 3320 | Accipiter | striatus | Bildstein, K. L., K. D. Meyer, C. M. White, J. S. Marks, & G. M. Kirwan (2020). Sharp-shinned Hawk (Accipiter striatus), version 1.0. In Birds of the World (S. M. Billerman, B. K. Keeney, P. G. Rodewald, & T. S. Schulenberg, Editors). Cornell Lab of Ornithology, Ithaca, NY, USA. |
| 3420 | Buteo | swainsoni | Bechard, M. J., C. S. Houston, J. H. Sarasola, & A. S. Engl& (2020). Swainson's Hawk (Buteo swainsoni), version 1.0. In Birds of the World (A. F. Poole, Editor). Cornell Lab of Ornithology, Ithaca, NY, USA. |
| 3430 | Buteo | platypterus | Goodrich, L. J., S. T. Crocoll, & S. E. Senner (2020). Broad-winged Hawk (Buteo platypterus), version 1.0. In Birds of the World (A. F. Poole, Editor). Cornell Lab of Ornithology, Ithaca, NY, USA. |
| 3480 | Buteo | regalis | Ng, J., M. D. Giovanni, M. J. Bechard, J. K. Schmutz, & P. Pyle (2020). Ferruginous Hawk (Buteo regalis), version 1.0. In Birds of the World (P. G. Rodewald, Editor). Cornell Lab of Ornithology, Ithaca, NY, USA. |
| 3490 | Aquila | chrysaetos | Katzner, T. E., M. N. Kochert, K. Steenhof, C. L. McIntyre, E. H. Craig, & T. A. Miller (2020). Golden Eagle (Aquila chrysaetos), version 2.0. In Birds of the World (P. G. Rodewald & B. K. Keeney, Editors). Cornell Lab of Ornithology, Ithaca, NY, USA. |
| 3520 | Haliaeetus | leucocephalus | Buehler, D. A. (2022). Bald Eagle (Haliaeetus leucocephalus), version 2.0. In Birds of the World (P. G. Rodewald & S. G. Mlodinow, Editors). Cornell Lab of Ornithology, Ithaca, NY, USA. |
| 3550 | Falco | mexicanus | Steenhof, K. (2020). Prairie Falcon (*Falco mexicanus*), version 1.0. In Birds of the World (A. F. Poole, Editor). Cornell Lab of Ornithology, Ithaca, NY, USA. |

| | | | |
|---|---|---|---|
| 3560 | Falco | peregrinus | White, C. M., N. J. Clum, T. J. Cade, & W. G. Hunt (2020). Peregrine Falcon (Falco peregrinus), version 1.0. In Birds of the World (S. M. Billerman, Editor). Cornell Lab of Ornithology, Ithaca, NY, USA. |
| 3600 | Falco | sparverius | Smallwood, J. A. & D. M. Bird (2020). American Kestrel (Falco sparverius), version 1.0. In Birds of the World (A. F. Poole & F. B. Gill, Editors). Cornell Lab of Ornithology, Ithaca, NY, USA. |
| 3640 | Pandion | haliaetus | Bierregaard, R. O., A. F. Poole, M. S. Martell, P. Pyle, & M. A. Patten (2020). Osprey (P&ion haliaetus), version 1.0. In Birds of the World (P. G. Rodewald, Editor). Cornell Lab of Ornithology, Ithaca, NY, USA. |
| 3670 | Asio | flammeus | Wiggins, D. A., D. W. Holt, & S. M. Leasure (2020). Short-eared Owl (Asio flammeus), version 1.0. In Birds of the World (S. M. Billerman, Editor). Cornell Lab of Ornithology, Ithaca, NY, USA. |
| 3900 | Megaceryle | alcyon | Kelly, J. F., E. S. Bridge, & M. J. Hamas (2020). Belted Kingfisher (Megaceryle alcyon), version 1.0. In Birds of the World (A. F. Poole, Editor). Cornell Lab of Ornithology, Ithaca, NY, USA. |
| 4160 | Antrostomus | carolinensis | Straight, C. A. & R. J. Cooper (2020). Chuck-will's-widow (Antrostomus carolinensis), version 1.0. In Birds of the World (A. F. Poole, Editor). Cornell Lab of Ornithology, Ithaca, NY, USA. |
| 4171 | Antrostomus | vociferus | Cink, C. L., P. Pyle, & M. A. Patten (2020). Eastern Whip-poor-will (Antrostomus vociferus), version 1.0. In Birds of the World (P. G. Rodewald, Editor). Cornell Lab of Ornithology, Ithaca, NY, USA. |
| 4210 | Chordeiles | acutipennis | Latta, S. C. & M. E. Baltz (2020). Lesser Nighthawk (Chordeiles acutipennis), version 1.0. In Birds of the World (A. F. Poole, Editor). Cornell Lab of Ornithology, Ithaca, NY, USA. |
| 4250 | Aeronautes | saxatalis | Ryan, T. P. & C. T. Collins (2020). White-throated Swift (Aeronautes saxatalis), version 1.0. In Birds of the World (A. F. Poole & F. B. Gill, Editors). Cornell Lab of Ornithology, Ithaca, NY, USA. |
| 4280 | Archilochus | colubris | Weidensaul, S., T. R. Robinson, R. R. Sargent, M. B. Sargent, & T. J. Zenzal (2020). Ruby-throated Hummingbird (Archilochus colubris), version 1.0. In Birds of the World (P. G. Rodewald, Editor). Cornell Lab of Ornithology, Ithaca, NY, USA. |
| 4290 | Archilochus | alexandri | Baltosser, W. H. & S. M. Russell (2020). Black-chinned Hummingbird (Archilochus alex&ri), version 1.0. In Birds of the World (A. F. Poole & F. B. Gill, Editors). Cornell Lab of Ornithology, Ithaca, NY, USA. |
| 4330 | Selasphorus | rufus | Healy, S. & W. A. Calder (2020). Rufous Hummingbird (Selasphorus rufus), version 1.0. In Birds of the World (A. F. Poole, Editor). Cornell Lab of Ornithology, Ithaca, NY, USA. |
| 4340 | Selasphorus | sasin | Clark, C. J. & D. E. Mitchell (2020). Allen's Hummingbird (Selasphorus sasin), version 1.0. In Birds of the World (A. F. Poole, Editor). Cornell Lab of Ornithology, Ithaca, NY, USA. |
| 4430 | Tyrannus | forficatus | Regosin, J. V. (2020). Scissor-tailed Flycatcher (*Tyrannus forficatus*), version 1.0. In Birds of the World (A. F. Poole, Editor). Cornell Lab of Ornithology, Ithaca, NY, USA. |
| 4470 | Tyrannus | verticalis | Gamble, L. R. & T. M. Bergin (2020). Western Kingbird (Tyrannus verticalis), version 1.0. In Birds of the World (A. F. Poole, Editor). Cornell Lab of Ornithology, Ithaca, NY, USA. |
| 4480 | Tyrannus | vociferans | Tweit, R. C. & J. C. Tweit (2020). Cassin's Kingbird (Tyrannus vociferans), version 1.0. In Birds of the World (A. F. Poole & F. B. Gill, Editors). Cornell Lab of Ornithology, Ithaca, NY, USA. |
| 4540 | Myiarchus | cinerascens | Cardiff, S. W. & D. L. Dittmann (2020). Ash-throated Flycatcher (Myiarchus cinerascens), version 1.0. In Birds of the World (A. F. Poole & F. B. Gill, Editors). Cornell Lab of Ornithology, Ithaca, NY, USA. |

| | | | |
|---|---|---|---|
| 5011 | Sturnella | neglecta | Davis, S. K. & W. E. Lanyon (2020). Western Meadowlark (Sturnella neglecta), version 1.0. In Birds of the World (A. F. Poole, Editor). Cornell Lab of Ornithology, Ithaca, NY, USA. |
| 5390 | Rhynchophanes | mccownii | With, K. A. (2021). Thick-billed Longspur (*Rhynchophanes mccownii*), version 1.1. In Birds of the World (A. F. Poole, Editor). Cornell Lab of Ornithology, Ithaca, NY, USA. |
| 5400 | Pooecetes | gramineus | Jones, S. L. & J. E. Cornely (2020). Vesper Sparrow (Pooecetes gramineus), version 1.0. In Birds of the World (A. F. Poole & F. B. Gill, Editors). Cornell Lab of Ornithology, Ithaca, NY, USA. |
| 5450 | Centronyx | bairdii | Green, M. T., P. E. Lowther, S. L. Jones, S. K. Davis, & B. C. Dale (2020). Baird's Sparrow (Centronyx bairdii), version 1.0. In Birds of the World (A. F. Poole & F. B. Gill, Editors). Cornell Lab of Ornithology, Ithaca, NY, USA. |
| 5480 | Ammospiza | leconteii | Lowther, P. E. (2020). LeConte's Sparrow (*Ammospiza leconteii*), version 1.0. In Birds of the World (P. G. Rodewald, Editor). Cornell Lab of Ornithology, Ithaca, NY, USA. |
| 5520 | Chondestes | grammacus | Martin, J. W. & J. R. Parrish (2020). Lark Sparrow (Chondestes grammacus), version 1.0. In Birds of the World (A. F. Poole & F. B. Gill, Editors). Cornell Lab of Ornithology, Ithaca, NY, USA. |
| 5540 | Zonotrichia | leucophrys | Chilton, G., M. C. Baker, C. D. Barrentine, & M. A. Cunningham (2020). White-crowned Sparrow (Zonotrichia leucophrys), version 1.0. In Birds of the World (A. F. Poole & F. B. Gill, Editors). Cornell Lab of Ornithology, Ithaca, NY, USA. |
| 5620 | Spizella | breweri | Rotenberry, J. T., M. A. Patten, & K. L. Preston (2020). Brewer's Sparrow (Spizella breweri), version 1.0. In Birds of the World (A. F. Poole & F. B. Gill, Editors). Cornell Lab of Ornithology, Ithaca, NY, USA. |
| 5650 | Spizella | atrogularis | Tenney, C. R. (2020). Black-chinned Sparrow (Spizella atrogularis), version 1.0. In Birds of the World (A. F. Poole & F. B. Gill, Editors). Cornell Lab of Ornithology, Ithaca, NY, USA. |
| 5730 | Amphispiza | bilineata | Johnson, M. J., C. van Riper, & K. M. Pearson (2020). Black-throated Sparrow (Amphispiza bilineata), version 1.0. In Birds of the World (A. F. Poole & F. B. Gill, Editors). Cornell Lab of Ornithology, Ithaca, NY, USA. |
| 5870 | Pipilo | erythrophthalmus | Greenlaw, J. S. (2020). Eastern Towhee (*Pipilo erythrophthalmus*), version 1.0. In Birds of the World (P. G. Rodewald, Editor). Cornell Lab of Ornithology, Ithaca, NY, USA. |
| 5900 | Pipilo | chlorurus | Dobbs, R. C., P. R. Martin, & T. E. Martin (2020). Green-tailed Towhee (Pipilo chlorurus), version 1.0. In Birds of the World (A. F. Poole, Editor). Cornell Lab of Ornithology, Ithaca, NY, USA. |
| 5950 | Pheucticus | ludovicianus | Wyatt, V. E. & C. M. Francis (2020). Rose-breasted Grosbeak (Pheucticus ludovicianus), version 1.0. In Birds of the World (A. F. Poole & F. B. Gill, Editors). Cornell Lab of Ornithology, Ithaca, NY, USA. |
| 5960 | Pheucticus | melanocephalus | Hill, G. E. (2022). Black-headed Grosbeak (Pheucticus melanocephalus), version 2.0. In Birds of the World (S. M. Billerman & B. K. Keeney, Editors). Cornell Lab of Ornithology, Ithaca, NY, USA. |
| 5970 | Passerina | caerulea | Lowther, P. E. & J. L. Ingold (2020). Blue Grosbeak (Passerina caerulea), version 1.0. In Birds of the World (A. F. Poole, Editor). Cornell Lab of Ornithology, Ithaca, NY, USA. |
| 6040 | Spiza | americana | Sousa, B. F., S. A. Temple, & G. D. Basili (2022). Dickcissel (Spiza americana), version 2.0. In Birds of the World (T. S. Schulenberg & B. K. Keeney, Editors). Cornell Lab of Ornithology, Ithaca, NY, USA. |
| 6050 | Calamospiza | melanocorys | Shane, T. G. (2020). Lark Bunting (Calamospiza melanocorys), version 1.0. In Birds of the World (A. F. Poole & F. B. Gill, Editors). Cornell Lab of Ornithology, Ithaca, NY, USA. |

| | | | |
|---|---|---|---|
| 6070 | Piranga | ludoviciana | Hudon, J. (2020). Western Tanager (Piranga ludoviciana), version 1.0. In Birds of the World (A. F. Poole & F. B. Gill, Editors). Cornell Lab of Ornithology, Ithaca, NY, USA. |
| 6170 | Stelgidopteryx | serripennis | De Jong, M. J. (2020). Northern Rough-winged Swallow (Stelgidopteryx serripennis), version 1.0. In Birds of the World (A. F. Poole & F. B. Gill, Editors). Cornell Lab of Ornithology, Ithaca, NY, USA. |
| 6200 | Phainopepla | nitens | Chu, M. & G. Walsberg (2020). Phainopepla (Phainopepla nitens), version 1.0. In Birds of the World (A. F. Poole & F. B. Gill, Editors). Cornell Lab of Ornithology, Ithaca, NY, USA. |
| 6220 | Lanius | ludovicianus | Yosef, R. (2020). Loggerhead Shrike (Lanius ludovicianus), version 1.0. In Birds of the World (A. F. Poole & F. B. Gill, Editors). Cornell Lab of Ornithology, Ithaca, NY, USA. |
| 6280 | Vireo | flavifrons | Rodewald, P. G. & R. D. James (2020). Yellow-throated Vireo (Vireo flavifrons), version 1.0. In Birds of the World (A. F. Poole, Editor). Cornell Lab of Ornithology, Ithaca, NY, USA. |
| 6450 | Leiothlypis | ruficapilla | Lowther, P. E. & J. M. Williams (2020). Nashville Warbler (Leiothlypis ruficapilla), version 1.0. In Birds of the World (A. F. Poole, Editor). Cornell Lab of Ornithology, Ithaca, NY, USA. |
| 6460 | Leiothlypis | celata | Gilbert, W. M., M. K. Sogge, & C. van Riper (2020). Orange-crowned Warbler (Leiothlypis celata), version 1.0. In Birds of the World (P. G. Rodewald, Editor). Cornell Lab of Ornithology, Ithaca, NY, USA. |
| 6470 | Leiothlypis | peregrina | Rimmer, C. C. & K. P. McFarl& (2020). Tennessee Warbler (Leiothlypis peregrina), version 1.0. In Birds of the World (A. F. Poole, Editor). Cornell Lab of Ornithology, Ithaca, NY, USA. |
| 6480 | Setophaga | americana | Moldenhauer, R. R. & D. J. Regelski (2020). Northern Parula (Setophaga americana), version 1.0. In Birds of the World (A. F. Poole, Editor). Cornell Lab of Ornithology, Ithaca, NY, USA. |
| 6680 | Setophaga | townsendi | Wright, A. L., G. D. Hayward, S. M. Matsuoka, & P. H. Hayward (2020). Townsend's Warbler (Setophaga townsendi), version 1.0. In Birds of the World (P. G. Rodewald, Editor). Cornell Lab of Ornithology, Ithaca, NY, USA. |
| 6690 | Setophaga | occidentalis | Pearson, S. F. (2020). Hermit Warbler (*Setophaga occidentalis*), version 1.0. In Birds of the World (A. F. Poole, Editor). Cornell Lab of Ornithology, Ithaca, NY, USA. |
| 6710 | Setophaga | pinus | Rodewald, P. G., J. H. Withgott, & K. G. Smith (2020). Pine Warbler (Setophaga pinus), version 1.0. In Birds of the World (A. F. Poole, Editor). Cornell Lab of Ornithology, Ithaca, NY, USA. |
| 6720 | Setophaga | palmarum | Wilson Jr., W. H. (2020). Palm Warbler (*Setophaga palmarum*), version 1.0. In Birds of the World (A. F. Poole, Editor). Cornell Lab of Ornithology, Ithaca, NY, USA. |
| 6730 | Setophaga | discolor | Nolan Jr, V., E. D. Ketterson, & C. A. Buerkle (2020). Prairie Warbler (Setophaga discolor), version 1.0. In Birds of the World (A. F. Poole, Editor). Cornell Lab of Ornithology, Ithaca, NY, USA. |
| 6750 | Parkesia | noveboracensis | Whitaker, D. M. & S. W. Eaton (2020). Northern Waterthrush (Parkesia noveboracensis), version 1.0. In Birds of the World (A. F. Poole, Editor). Cornell Lab of Ornithology, Ithaca, NY, USA. |
| 6810 | Geothlypis | trichas | Guzy, M. J. & G. Ritchison (2020). Common Yellowthroat (Geothlypis trichas), version 1.0. In Birds of the World (P. G. Rodewald, Editor). Cornell Lab of Ornithology, Ithaca, NY, USA. |
| 6830 | Icteria | virens | Thompson, C. F. & K. P. Eckerle (2022). Yellow-breasted Chat (Icteria virens), version 2.0. In Birds of the World (P. G. Rodewald & B. K. Keeney, Editors). Cornell Lab of Ornithology, Ithaca, NY, USA. |

| | | | |
|---|---|---|---|
| 6850 | Cardellina | pusilla | Ammon, E. M. & W. M. Gilbert (2020). Wilson's Warbler (Cardellina pusilla), version 1.0. In Birds of the World (P. G. Rodewald, Editor). Cornell Lab of Ornithology, Ithaca, NY, USA. |
| 7000 | Anthus | spragueii | Davis, S. K., M. B. Robbins, & B. C. Dale (2020). Sprague's Pipit (Anthus spragueii), version 1.0. In Birds of the World (A. F. Poole, Editor). Cornell Lab of Ornithology, Ithaca, NY, USA. |
| 7050 | Toxostoma | rufum | Cavitt, J. F. & C. A. Haas (2020). Brown Thrasher (Toxostoma rufum), version 1.0. In Birds of the World (A. F. Poole, Editor). Cornell Lab of Ornithology, Ithaca, NY, USA. |
| 7080 | Toxostoma | bendirei | Engl&, A. S. & W. F. Laudenslayer Jr. (2020). Bendire's Thrasher (Toxostoma bendirei), version 1.0. In Birds of the World (A. F. Poole & F. B. Gill, Editors). Cornell Lab of Ornithology, Ithaca, NY, USA. |
| 7190 | Thryomanes | bewickii | Kennedy, E. D. & D. W. White (2020). Bewick's Wren (Thryomanes bewickii), version 1.0. In Birds of the World (A. F. Poole, Editor). Cornell Lab of Ornithology, Ithaca, NY, USA. |
| 7260 | Certhia | americana | Poulin, J., É. D'Astous, M. Villard, S. J. Hejl, K. R. Newlon, M. E. McFadzen, J. S. Young, & C. K. Ghalambor (2020). Brown Creeper (Certhia americana), version 1.0. In Birds of the World (A. F. Poole, Editor). Cornell Lab of Ornithology, Ithaca, NY, USA. |
| 7490 | Corthylio | calendula | Swanson, D. L., J. L. Ingold, & G. E. Wallace (2021). Ruby-crowned Kinglet (Corthylio calendula), version 1.1. In Birds of the World (Editor not available). Cornell Lab of Ornithology, Ithaca, NY, USA. |
| 7540 | Myadestes | townsendi | Bowen, R. V. (2020). Townsend's Solitaire (Myadestes townsendi), version 1.0. In Birds of the World (A. F. Poole & F. B. Gill, Editors). Cornell Lab of Ornithology, Ithaca, NY, USA. |
| 7550 | Hylocichla | mustelina | Evans, M., E. Gow, R. R. Roth, M. S. Johnson, & T. J. Underwood (2020). Wood Thrush (Hylocichla mustelina), version 1.0. In Birds of the World (A. F. Poole, Editor). Cornell Lab of Ornithology, Ithaca, NY, USA. |
| 7610 | Turdus | migratorius | V&erhoff, N., P. Pyle, M. A. Patten, R. Sallabanks, & F. C. James (2020). American Robin (Turdus migratorius), version 1.0. In Birds of the World (P. G. Rodewald, Editor). Cornell Lab of Ornithology, Ithaca, NY, USA. |
